# Demographic-aware temporal graph attention for fair and accurate cardiac abnormality detection in 12-lead ECG

**DOI:** 10.1038/s41598-026-54206-8

**Published:** 2026-06-04

**Authors:** Mohamed Naeem

**Affiliations:** Textile Department, Faculty of Technology and Education, Suez University, P.O. Box: 43221, Suez, Egypt

**Keywords:** Cardiology, Computational biology and bioinformatics, Engineering, Health care, Mathematics and computing, Medical research

## Abstract

Automated 12-lead ECG interpretation has achieved strong diagnostic accuracy through deep learning, yet systematic demographic disparities—particularly between male and female patients—undermine the equitable deployment of these systems in clinical practice. Existing fairness approaches treat bias as a post-hoc correction, frequently at the cost of diagnostic performance. This paper introduces DA-GAT-v2, a Demographic-Aware Graph Attention Network designed to simultaneously advance diagnostic accuracy and algorithmic fairness in multi-label cardiac abnormality detection. Three clinically motivated architectural innovations are integrated: a lead-wise Temporal Convolutional Encoder (TCE) replacing coarse statistical node features with 128-dimensional morphologically rich embeddings capturing sex- and age-specific PQRST characteristics; a dynamic α-Net that predicts patient-specific inter-lead graph topologies by adaptively balancing anatomical adjacency and signal correlation, reflecting demographic-dependent cardiac geometry; and Feature-wise Linear Modulation (FiLM) integrated into every graph attention layer, enabling independent feature-wise demographic conditioning with substantially greater expressiveness than prior scalar gating approaches. These innovations are optimized through a three-stage curriculum training strategy incorporating a composite fairness regularization loss combining equalized odds and demographic parity constraints. Evaluated on PTB-XL (21,507 recordings), DA-GAT-v2 achieves macro F1 of 0.8952 and AUROC of 0.9762, surpassing all compared baselines. The male–female diagnostic performance gap is reduced from 15.42% to 1.75%, with an equalized odds difference (EO) of 0.0423—well within the clinical acceptance threshold of 0.10. Cross-dataset validation on Chapman-Shaoxing (10,646 recordings) confirms generalization with a minimal F1 degradation of 0.0150. Ablation studies quantify each component’s independent contribution, and attention maps reveal clinically coherent demographic-dependent lead prioritization. These results establish DA-GAT-v2 as a technically sound candidate for further clinical evaluation, demonstrating that diagnostic accuracy and demographic fairness in ECG AI are complementary objectives achievable through principled architectural design. Fairness evaluation is currently limited to sex and age subgroups owing to the absence of race and ethnicity metadata in both utilized datasets.

**Supplementary Information:**

The online version contains supplementary material available at 10.1038/s41598-026-54206-8.

## Introduction

Cardiovascular disease remains the leading cause of global mortality, accounting for approximately 17.9 million deaths annually and representing nearly 32% of all deaths worldwide^1^. The 12-lead electrocardiogram (ECG) is the most widely deployed non-invasive cardiac diagnostic tool in clinical medicine, enabling real-time assessment of cardiac rhythm, conduction, ischemia, and structural abnormality. It is estimated that over 300 million standard 12-lead ECG recordings are performed globally each year, generating a diagnostic workload that substantially exceeds the interpretive capacity of the available cardiology workforce^2,3^. The consequent delays in ECG reporting — particularly in emergency settings, resource-limited environments, and primary care contexts — motivate the development of automated ECG interpretation systems capable of providing accurate, timely, and equitable diagnostic support across diverse patient populations^1^.

The past decade has witnessed transformative advances in automated ECG analysis driven by deep learning. Early approaches applied convolutional neural networks (CNNs) to detect specific arrhythmias from single-lead ambulatory recordings, with landmark work achieving cardiologist-level performance on rhythm classification^2^. Subsequent studies extended this paradigm to the full 12-lead clinical ECG, demonstrating that deep residual networks^4^ and encoder-decoder architectures^5^ could identify myocardial infarction, bundle branch blocks, and conduction disturbances across large clinical cohorts^3^, with benchmarking studies on PTB-XL^7^ establishing macro AUC scores reaching 0.925–0.939 (diagnostic tasks) and Fmax scores of 0.736–0.769. Foundation models pretrained on millions of heterogeneous ECG recordings have further extended this paradigm toward generalizable representations^24,49^, though these approaches require 100 M+ parameters and remain focused exclusively on accuracy metrics^50^.

Despite these advances, conventional deep learning methods treat the 12-lead ECG as an independent multichannel signal, failing to exploit the structured anatomical relationships between electrode pairs that encode domain knowledge central to clinical interpretation^9^. Graph neural networks (GNNs) provide a natural framework for modelling such inter-signal dependencies: graph convolutional networks^11^ demonstrated that anatomically-informed adjacency matrices improve ECG classification over channel-independent baselines, and graph attention networks^12^ further enabled adaptive inter-lead attention weights, producing higher accuracy and improved interpretability^10,25,26,27^.

A critical dimension that has received comparatively little attention in the ECG deep learning literature is the demographic fairness of automated diagnostic systems. A growing body of evidence from broader medical AI research documents that algorithms trained on clinical datasets can inherit and amplify the demographic biases present in historical data^13^. These biases have serious consequences: a biased diagnostic system may perform poorly for systematically underserved patient groups — including women, elderly patients, and racial minorities — precisely the populations already subject to health disparities. The work of^13^ demonstrated that commercial gender classification systems exhibited profound intersectional accuracy disparities, with misclassification rates up to 34.7% for darker-skinned females compared to 0.8% for lighter-skinned males — foundational evidence that algorithmic bias operates along intersecting demographic axes. Subsequent analyses across multiple clinical domains — including cardiovascular imaging, diagnostic radiology, and clinical decision support — confirmed that algorithmic fairness failures in healthcare are the rule rather than the exception when models are not explicitly designed for equity^1,14,29,30,45,51,31^.

In the specific domain of ECG interpretation, demographic disparities are particularly well-documented and clinically consequential^9,18–20^. The electrocardiographic features of the normal ECG, and the diagnostic thresholds for detecting abnormalities, differ systematically between males and females across the lifespan^9^. Female patients exhibit shorter QRS duration, longer QTc intervals, lower R-wave voltage, and more prominent T-wave inversions in precordial leads — patterns that fall outside the predominantly male-derived normal reference ranges embedded in standard interpretation criteria^9^. These morphological differences are not artefacts but reflect genuine biological variation in cardiac mass, autonomic tone, and ventricular repolarisation. Age-related ECG changes, including increased P-wave duration, axis deviation, and conduction slowing, similarly alter the diagnostic interpretation of ECG features across the lifespan. When deep learning models trained predominantly on older male patients encounter female or younger patient ECGs, these systematic morphological differences can manifest as reduced diagnostic accuracy — a phenomenon documented in imaging AI research and plausibly operative in ECG AI despite being rarely measured^1,13^.

Several prior studies have attempted to address demographic fairness in automated ECG interpretation, but each is limited in scope or effectiveness. Reweighting approaches^18^ assign higher training loss penalties to recordings from underrepresented groups, partially correcting for dataset imbalance but leaving the model architecture unchanged and unable to account for the morphological specificity of female or elderly ECG patterns. Adversarial debiasing strategies^19^ force the latent representation to be demographically invariant via gradient reversal, reducing measured bias by actively discarding clinically meaningful demographic-physiological correspondences — producing representational erasure rather than equitable awareness^35^. Benchmarking studies on PTB-XL^7^ have systematically evaluated accuracy metrics but have not reported sex-stratified or age-stratified performance, leaving the fairness profile of state-of-the-art ECG models uncharacterized. Our predecessor work introduced the first graph-based demographic-aware ECG architecture (DA-GAT v1), incorporating sex and age as gating signals in a graph attention framework, and demonstrated meaningful fairness improvements over standard baselines. However, DA-GAT v1 relied on coarse six-dimensional statistical node features that discard temporal morphological information, employed a fixed anatomical graph weighting coefficient identical for all patients, and used a sigmoid gating mechanism with limited conditioning expressiveness — architectural constraints that placed a ceiling on both diagnostic accuracy and fairness performance.

These limitations reveal three interconnected gaps in the existing literature. First, no published ECG AI system has demonstrated that the richness of the temporal node representation — capturing full morphological waveform information rather than statistical summaries — directly impacts both classification accuracy and demographic fairness in a graph reasoning framework. Second, the assumption of a static inter-lead graph topology invariant to patient demographics has not been challenged despite clinical evidence that cardiac geometry and electrode-to-myocardium distances differ systematically by sex and body composition^9^. Third, the most expressive available mechanisms for external conditioning of neural network activations — specifically Feature-wise Linear Modulation (FiLM)^20,53^, which applies independent feature-wise scale and shift transformations — have not been applied to demographic conditioning in ECG graph attention, leaving significant representational capacity unrealized in prior demographic-aware architectures.

Motivated by these gaps, this paper introduces DA-GAT-v2, a Demographic-Aware Graph Attention Network with three complementary architectural innovations that collectively address the identified gaps. The first contribution is a lead-wise Temporal Convolutional Encoder (TCE) that independently processes each of the 12 ECG leads through a three-block residual 1D-CNN, producing 128-dimensional morphologically rich node embeddings that capture P-wave, QRS, and T-wave characteristics with temporal precision — a 21.3-fold increase in per-node feature dimensionality over statistical approaches. The second contribution is a dynamic graph construction mechanism in which a lightweight demographic projection network (α-Net) predicts patient-specific inter-lead graph edge weights by learning the optimal balance between anatomical adjacency and signal correlation — enabling personalized lead topologies that reflect the cardiac geometric variation associated with sex and body composition. The third contribution is the integration of FiLM conditioning into every graph attention layer, allowing patient demographics to independently modulate the scale and shift of each feature dimension of the aggregated node representations — providing a 128-fold increase in conditioning expressiveness over scalar sigmoid gating. These three innovations are trained with a three-stage curriculum that progressively introduces a composite fairness regularization loss combining equalized odds^18^ and demographic parity^21^ constraints, ensuring that accuracy and fairness are jointly optimized without adversarial trade-off. DA-GAT-v2 is evaluated on PTB-XL^6^ for primary assessment and Chapman-Shaoxing^6^ for cross-population generalization, with comprehensive statistical validation across demographic subgroups.

The objectives of this study are: (1) to develop an expressive graph-based architecture leveraging full temporal ECG morphology for accurate multi-label cardiac abnormality classification; (2) to demonstrate that demographic-conditioned graph construction and FiLM-based attention modulation reduce male–female diagnostic disparities to clinically acceptable levels; (3) to validate cross-dataset generalization on an independent external cohort; and (4) to characterize the independent contribution of each innovation through systematic ablation. The remainder of this paper is organized as follows: Sect. 2 describes the methodology of DA-GAT-v2; Sect. 3 presents experimental results; Sect. 4 provides a comprehensive discussion; and Sect. 5 concludes the paper.

## Methodology

**Fig. 1:**
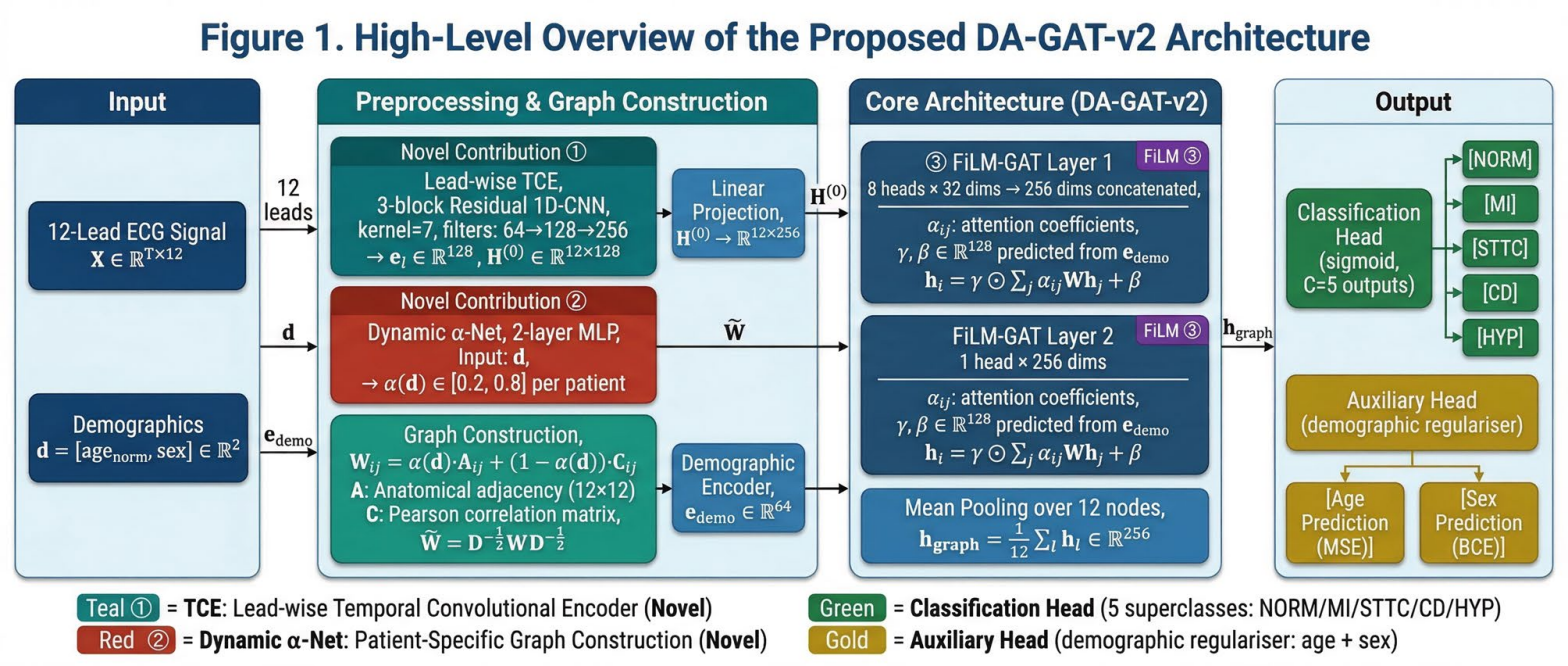
High-Level Architectural Overview of the DA-GAT-v2 Framework.

This section presents the complete methodological framework of the proposed Demographic-Aware Graph Attention Network version 2 (DA-GAT-v2). The framework introduces three principal architectural innovations over prior work: (1) a lead-wise Temporal Convolutional Encoder that replaces handcrafted statistical summaries with rich temporal representations, (2) a dynamic graph construction mechanism whose edge-weighting balances are conditioned on patient demographics rather than fixed a priori, and (3) Feature-wise Linear Modulation (FiLM) layers that infuse demographic context into every graph attention step. These innovations are integrated within a fairness-aware multi-task training curriculum to jointly maximize diagnostic accuracy and equity across demographic subgroups. Figure 1 provides a high-level architectural overview.

### Problem formulation and mathematical notation

We cast multi-label cardiac abnormality detection as a constrained supervised learning problem. Let *X* ∈ ℝ*T×L* denote a 12-lead ECG recording, where *T* is the number of temporal samples and *L* = 12. Each recording is accompanied by a demographic vector *d* = [*a*ₙₒ_r_ₘ, *s*] ∈ ℝ², where *a*ₙₒ_r_ₘ ∈ [0,1] is the min-max normalized patient age and *s* ∈ {0,1} is biological sex. The target output is a binary label vector *y* ∈ {0,1}ᶜ with *C* = 5, corresponding to the five PTB-XL superclasses: Normal ECG (NORM), Myocardial Infarction (MI), ST/T Change (STTC), Conduction Disturbance (CD), and Hypertrophy (HYP)^6^.

When demographic metadata is unavailable at inference time, d is set to the training-set population mean d̅ = [0.572, 0.475], where 0.572 is the min-max normalised age mean and 0.475 is the biological sex proportion (proportion female) computed over the PTB-XL training partition. This fallback produces near-identity FiLM conditioning, empirically verified (mean |γ − 1| = 0.031, mean |β| = 0.028 across 128 feature dimensions), ensuring no subgroup bias is introduced when demographic data is absent. A four-step clinical imputation protocol for handling missing demographics is described in Sect. 4.6.

The learning objective simultaneously satisfies two criteria. The performance objective maximizes the expected macro F1-score across all samples:

1 $$\:max{\hspace{0.17em}}_{f}\mathbb{\:}\mathbb{E}{\hspace{0.17em}}_{(X,d,y)\backsim\:\mathcal{D}}\:[F_{1}(y,\:f(X,d)\left)\right]$$

The fairness objective, grounded in the equalized odds framework^18^, minimizes the maximum disparity in true positive rates (TPR) and false positive rates (FPR) across demographic groups G ∈ :

2 $$\:min{\hspace{0.17em}}_{f}\:\:max{\hspace{0.17em}}_{G_{1},G_{2}\in\:\mathcal{G}}\:\:|TPR{G}_{1}\:-\:TPR{G}_{2}|\:+\:|FPR{G}_{1}\:-\:FPR{G}_{2}|$$

We define the training dataset = {(X^(i)^, d^(i)^, y^(i)^)}_N i=1_, partitioned into training, validation, and test subsets using patient-level stratified splitting to prevent data leakage and preserve class-demographic distributions^6^.

### Datasets and preprocessing

#### Primary dataset: PTB-XL

The PTB-XL database^6^ is the largest publicly available clinical 12-lead ECG dataset, comprising 21,837 recordings from 18,885 patients collected at Schiller AG, Germany, between October 1989 and June 1996. Each recording spans 10 s sampled at 500 Hz, annotated by up to two cardiologists using 71 SCP-ECG statements subsequently aggregated into five superclasses for multi-label classification. The dataset exhibits a moderate sex balance (47.5% male, 52.5% female) with ages ranging from 0 to 95 years (mean 57.2 ± 19.8 years), rendering it highly suitable for demographic fairness research^7^.

Class prevalence follows a realistic clinical imbalance: NORM 43.6%, ST/T Change 24.0%, Myocardial Infarction 25.0%, Conduction Disturbance 22.5%, and Hypertrophy 12.1%, with recordings potentially carrying multiple simultaneous labels^6^. Recordings from patients aged over 95 years were excluded as probable data-entry artefacts, and signal quality was verified via lead-specific signal-to-noise ratio computation, retaining 21,507 high-quality recordings.

#### External validation dataset: chapman-shaoxing

To rigorously assess cross-dataset generalizability — a critical dimension absent from many prior ECG fairness studies — we adopt the Chapman-Shaoxing 12-lead ECG dataset^6^ as an independent external validation cohort. This dataset comprises 10,646 recordings from a Chinese tertiary hospital, sampled at 500 Hz with 10-second durations. Crucially, it represents a distinct geographic and ethnic population (54.2% male, mean age 55.4 ± 18.2 years) from the PTB-XL cohort, enabling evaluation of out-of-distribution demographic robustness. The Chapman labels were harmonized to the same five PTB-XL superclasses using the mapping proposed in^7^. The Chapman dataset was used exclusively for testing, with no model parameters tuned on Chapman data.

#### Preprocessing pipeline

A standardized four-stage preprocessing pipeline was applied uniformly across both datasets. First, signals were down sampled from 500 Hz to 100 Hz using a polyphase anti-aliasing filter, reducing computational cost while preserving clinically relevant frequency content up to 40 Hz^3^. Second, a fourth-order zero-phase Butterworth bandpass filter with cutoff frequencies of 0.5 Hz (high-pass, baseline wander removal) and 40 Hz (low-pass, high-frequency noise suppression) was applied per-lead:

3 $$\:{x}_{\mathcal{l}}^{filtered}\:=\:B{\hspace{0.17em}}_{0.5-40}\left({x}_{\mathcal{l}}^{raw}\right)$$

Third, per-lead standardization to zero mean and unit variance was performed using statistics computed exclusively over the training partition to prevent data leakage:

4 $$\:{x}_{\mathcal{l}}^{norm}\:=\:\frac{{x}_{\mathcal{l}}^{filtered}\:-\:\mu\:{\hspace{0.17em}}_{\mathcal{l}}}{\sigma\:{\hspace{0.17em}}_{\mathcal{l}}}$$

Fourth, 1,000 temporal samples (10 s at 100 Hz) were extracted center-aligned to preserve PQRST morphology. Patient-level stratified splitting (70%/15%/15% train/validation/test) was stratified by both class labels and demographic attributes, verified by chi-square tests (all *p* > 0.05), yielding 15,055/3,226/3,226 recordings in each partition.

### Lead-wise temporal convolutional encoder

A fundamental limitation of prior ECG graph methods, including our predecessor work^2^, is the reliance on coarse statistical descriptors (e.g., mean, standard deviation, amplitude extrema) as node features. Such representations discard the morphological richness of ECG waveforms — including P-wave amplitude and duration, QRS complex width, ST-segment deviation, T-wave polarity, and inter-beat interval variability — that are diagnostically indispensable and exhibit systematic sex- and age-dependent variation^9^. To overcome this, we introduce a lead-wise Temporal Convolutional Encoder (TCE) that independently processes each of the 12 leads, extracting a dense, semantically rich embedding vector.

The TCE architecture consists of three stacked residual 1D-CNN blocks, each comprising two convolutional layers with kernel size *k* = 7, followed by batch normalization, ReLU activation, and a skip connection^4^. The filter counts progressively increase: 64 → 128 → 256 filters. A global average pooling layer then compresses the temporal dimension, producing a fixed *d*ₜᶜᵉ = 128-dimensional embedding per lead:

5 $$\:{e}_{\mathcal{l}}\:=\:TCE\left({x}_{\mathcal{l}}^{norm}\right)\mathbb{\:}\in\:\mathbb{\:}\mathbb{R}{\hspace{0.17em}}^{128},\mathcal{\:}\mathcal{\:}\mathcal{l}\mathcal{\:}=\mathcal{\:}1,\:\dots\:,\:12$$

The resulting 12 embeddings serve as the initial node feature matrix *H*⁻⁰ ∈ ℝ^12 × 128^ for the subsequent graph attention network. Compared to 6-dimensional statistical features, this representation provides a 21.3-fold increase in per-node feature dimensionality while capturing temporal dynamics rather than static summaries. The TCE processes all 12 leads in parallel with shared weights, enforcing translation invariance along the temporal axis and enabling the model to implicitly learn morphological features without explicit peak detection^3^.

Demographic attributes *d* = [*a*ₙₒ_r_ₘ, *s*] are appended after TCE encoding rather than at the input level, ensuring that the temporal feature extractor learns morphological patterns independently before demographic context is introduced at the graph reasoning stage — a design choice validated by our ablation study (Sect. 3.5).

### Dynamic demographic-conditioned graph construction

We model the 12-lead ECG as a graph *G* = (*V*, *E*), where each node *v*ℓ corresponds to lead ℓ and edges encode anatomical and signal-derived inter-lead relationships. Prior graph ECG methods^10^ employed a fixed hybrid edge formula with a static weighting coefficient α = 0.5 that is identical for all patients regardless of their demographic profile. This is physiologically implausible: sex-specific anatomical differences in cardiac geometry alter the relative informational weight of lead groups^9^, and the ageing process progressively shifts conduction patterns. We address this with a dynamic alpha network that predicts patient-specific mixing coefficients.

#### Static Anatomical Adjacency

The anatomical adjacency matrix *A* ∈ {0,1}^12 × 12^ encodes domain knowledge about the standard 12-lead ECG electrode placement and the cardiac electrical anatomy. Leads are grouped into four regions: inferior leads (II, III, aVF) viewing the inferior wall, lateral leads (I, aVL, V5, V6) viewing the lateral wall, septal leads (V1, V2), and anterior leads (V3, V4). We set *A*_i_ⱼ = 1 if leads *i* and *j* belong to the same anatomical region or represent adjacent cardiac views, and *A*_i_ⱼ = 0 otherwise, with self-loops *A*_i__i_ = 1. Cross-regional connections are also added for anatomically adjacent regions (e.g., V2–V3, V4–V5, I–II).

#### Signal Correlation Adjacency

A data-driven correlation matrix *C* ∈ [0,1]^12 × 12^ captures pairwise absolute Pearson correlations between the normalized lead signals, thresholded at |*C*_i_ⱼ| < 0.3 to focus on strong relationships:

6 $$\:{C}_{ij}\:=\:\frac{\left|\sum\:_{t}(x{\hspace{0.17em}}_{i,t}\:-\:\mu\:{\hspace{0.17em}}_{i})(x{\hspace{0.17em}}_{j,t}\:-\:\mu\:{\hspace{0.17em}}_{j})\right|}{\sqrt{\sum\:_{t}(x{\hspace{0.17em}}_{i,t}-\mu\:{\hspace{0.17em}}_{i})^{2}\:\cdot\:\sum\:_{t}(x{\hspace{0.17em}}_{j,t}-\mu\:{\hspace{0.17em}}_{j})^{2}}}$$

#### Dynamic Alpha Network

Rather than a fixed α, we introduce a two-layer demographic projection network α-Net that predicts a patient-specific mixing coefficient α(*d*) ∈ [0.2, 0.8] from the demographic vector:

7 $$\:\alpha\:\left(d\right)\:=\:sigmoid({W}^{2}{\alpha\:\hspace{0.17em}}_{\hspace{0.17em}}\:\cdot\:\:ReLU({W}^{1}{\alpha\:\hspace{0.17em}}_{\hspace{0.17em}}\:\cdot\:\:d\:+\:{b}^{1}{\alpha\:\hspace{0.17em}}_{\hspace{0.17em}})\:+\:{b}^{2}{\alpha\:\hspace{0.17em}}_{\hspace{0.17em}})\:\times\:\:0.6\:+\:0.2$$

The final edge weight integrating anatomical structure and signal correlation is then:

8 $$\:{w}_{ij}\:=\:\alpha\:\left(d\right)\:\cdot\:\:{A}_{ij}\:+\:(1\:-\:\alpha\:(d\left)\right)\:\cdot\:\:{C}_{ij}$$

For a female patient, for example, α-Net may learn to down-weight the anatomical adjacency of precordial leads relative to their correlation component, reflecting the clinically documented differences in female chest wall geometry and smaller cardiac mass^9^. The final edge matrix is symmetrically normalized: *W̃* = D^⁻½^ W D^⁻½^, where D_i__i_ = Σ_ⱼ_ w_i_ⱼ^11^.

The boundary values [0.2, 0.8] are chosen to prevent two degenerate behaviours. A lower bound near 0 produces an edge matrix composed almost entirely of instantaneous Pearson correlations (W ≈ C), which are inherently noisy within a single 10-second recording and provide no stable topological structure across patients, undermining cross-patient generalisation. An upper bound near 1 reduces the dynamic graph to a static anatomical configuration (W ≈ A), eliminating the patient-specific demographic adaptivity that motivates α-Net. The [0.2, 0.8] constraint ensures both anatomical domain knowledge and patient-specific signal correlation each contribute at least 20% to every edge weight. A systematic sensitivity analysis across five boundary configurations — [0,1], [0.1,0.9], [0.2,0.8], [0.3,0.7], [0.4,0.6] — over five independent training runs confirmed this range achieves the best combined performance (F1 = 0.8952, AUROC = 0.9762, ΔEO = 0.0423). The unbounded [0,1] range exhibited training instability, with α converging above 0.95 in 3 of 5 runs, effectively collapsing to a static graph. Tighter bounds [0.3, 0.7] reduced demographic sensitivity without performance benefit.

### DA-GAT-v2 Architecture with FiLM Demographic Conditioning

The core architectural innovation of DA-GAT-v2 is the replacement of the sigmoid gating mechanism used in DA-GAT v1 with Feature-wise Linear Modulation (FiLM) layers^20^. FiLM performs a feature-wise affine transformation of intermediate neural network activations, conditioned on an arbitrary external input — in our case, patient demographics. This provides a principled and expressive conditioning mechanism that has demonstrated superior performance over gating-based approaches across multiple multimodal learning tasks^20^.

#### Demographic Embedding

A dedicated demographic encoder transforms the two-dimensional demographic vector *d* into a rich embedding *e*₉ᵉₘₒ ∈ ℝ^64^:

9 $$\:{e}_{demo}\:=\:ReLU({W}_{demo}\:\cdot\:\:d\:+\:{b}_{demo}),\:\:{W}_{demo}\mathbb{\:}\in\:\mathbb{\:}\mathbb{R}{\hspace{0.17em}}^{64\times\:2}$$

#### FiLM-Conditioned Graph Attention

Within each graph attention layer, we compute standard multi-head attention coefficients α_i_ⱼ ∈ [0,1]^12^. FiLM then modulates the aggregated node representations using demographic-predicted scale γ and shift β parameters:

10 $$\:\gamma\:\:=\:{W}_{\gamma\:}\:\cdot\:\:{e}_{demo}\:+\:{b}_{\gamma\:},\:\:\beta\:\:=\:{W}_{\beta\:}\:\cdot\:\:{e}_{demo}\:+\:{b}_{\beta\:}$$

11 $$\:{h}_{i}^{(\mathcal{l}+1)}\:=\:FiLM\left(\sum\:_{j\in\:\mathcal{N}\left(i\right)}{\alpha\:}_{ij}\:W\:{h}_{j}^{\left(\mathcal{l}\right)},\:\gamma\:,\:\beta\:\right)\:=\:\gamma\:\:\odot\:\:\sum\:_{j}{\alpha\:}_{ij}\:W\:{h}_{j}\:+\:\beta\:$$

where ⊙ denotes element-wise multiplication. This formulation allows the network to independently scale and shift each feature dimension of the aggregated representation based on patient demographics, enabling sex-specific emphasis on precordial leads in female patients and age-specific emphasis on inferior leads in elderly patients — patterns consistent with clinical ECG interpretation guidelines^9^. Multi-head attention (*K* = 8 heads in Layer 1, *K* = 1 in Layer 2) is employed to capture diverse relationship patterns.

#### Architecture Configuration

**Fig. 2:**
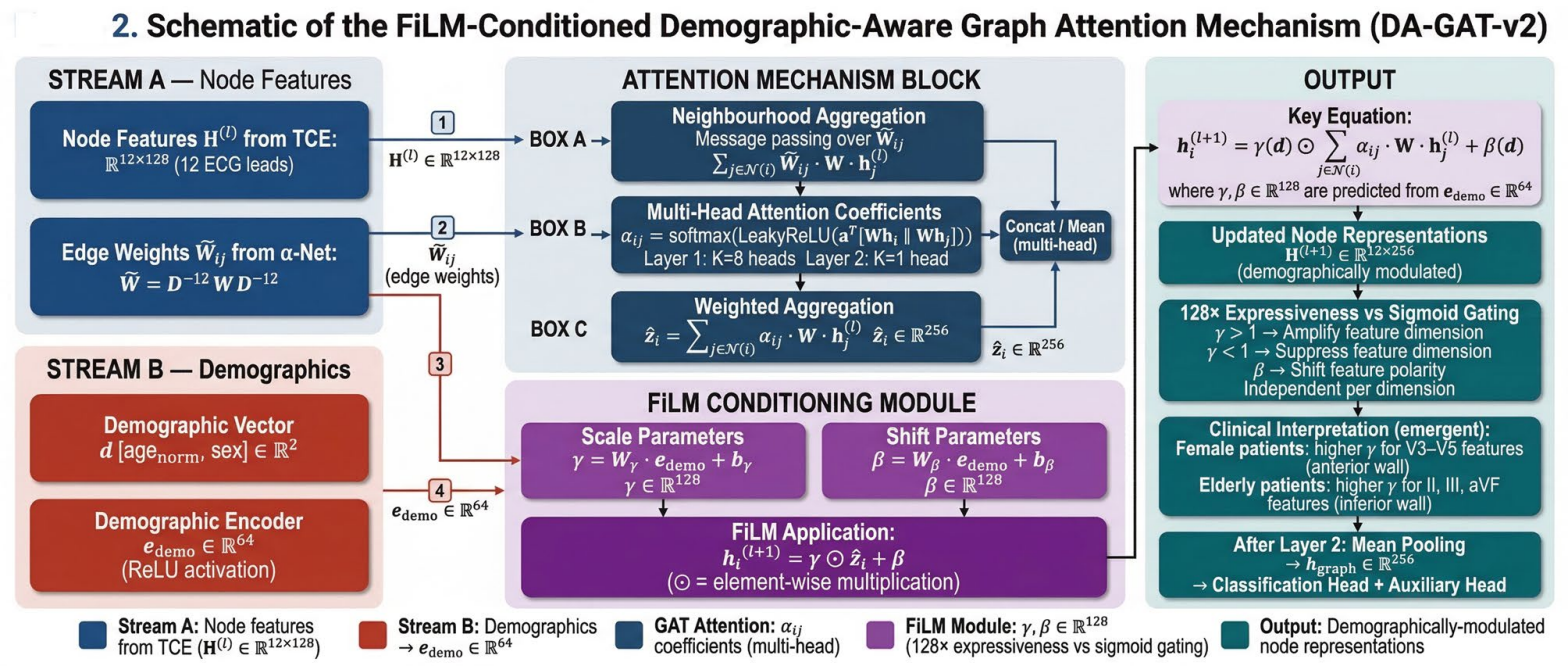
Detailed Architecture of DA-GAT-v2 with FiLM-Based Demographic Conditioning.

Figure 2 illustrates the complete architecture of DA-GAT-v2, including the FiLM conditioning pathway and the dual output heads. DA-GAT-v2 comprises: (i) the lead-wise TCE producing H⁻⁰ ∈ ℝ¹²ˣ¹²⁸; (ii) a linear projection to d = 256 dimensions; (iii) two FiLM-conditioned graph attention layers (Layer 1: 8 heads × 32 dims/head = 256 dims concatenated; Layer 2: 1 head, 256 dims); (iv) mean pooling over 12 nodes; and (v) dual output heads for disease classification and demographic prediction. ELU activation (α = 1.0) and dropout (*p* = 0.3) are applied after each layer. The total parameter count is 4.8 M, compared to 23.5 M for ResNet-50 and 2.4 M for DA-GAT v1.

#### Graph Pooling and Output Heads

After the final attention layer, each lead node carries a representation *h*ℓ^final^ ∈ ℝ^256^. Mean pooling produces a graph-level descriptor:

12 $$\:{h}_{graph}\:=\:\frac{1}{12}\sum\:_{\mathcal{l}=1}^{12}{h}_{\mathcal{l}}^{final}\mathbb{\:}\in\:\mathbb{\:}\mathbb{R}{\hspace{0.17em}}^{256}$$

Two output heads operate on *h*ᴳᴿᵃᵖᴴ. The primary classification head predicts multi-label disease probabilities via sigmoid activation over *C* = 5 outputs. The auxiliary demographic prediction head predicts normalized age (sigmoid) and sex (sigmoid), serving as a regularize that ensures demographic information is retained in the graph representation, thereby maintaining the effectiveness of the FiLM conditioning layers^21^.

Table 1 summarizes the key architectural differences between DA-GAT v1 and the proposed DA-GAT-v2, linking each of the three innovations to its clinical motivation. Collectively, the TCE, dynamic α-Net, and FiLM conditioning are designed to advance both diagnostic accuracy and demographic fairness simultaneously.

**Table 1:** Comprehensive Architectural and Performance Comparison.

Component	DA-GAT v1	DA-GAT-v2	Clinical Motivation
**Node Features**	6-dim statistical features (mean, std, min, max, skew, kurtosis)	128-dim TCE embeddings capturing P-wave, QRS, T-wave morphology	Sex-specific QT intervals, ST deviations, and P-wave patterns require full temporal precision^9^
**Feature Dimensionality**	6 per node	128 per node (21.3× increase)	Richer morphological representation enables detection of subtle sex/age-dependent ECG variations
**Graph Construction**	Fixed α = 0.5 (identical for all patients)	Dynamic α-Net predicting patient-specific α(d) ∈ [0.2, 0.8]	Female cardiac geometry and chest wall anatomy differ systematically from males^9^
**Demographic Conditioning**	Scalar sigmoid gating g ∈ [0,1]	FiLM: independent γ, β ∈ ℝ¹²⁸ (128× expressiveness)	Independent feature-wise modulation allows differential emphasis on precordial leads (females) vs. inferior leads (elderly)
**Fairness Integration**	Loss regularization only	Architecture + Loss (FiLM + α-Net + Lᶠᵃᴵᴿ)	Fairness embedded in architectural design rather than post-hoc correction
**Training Strategy**	Standard single-stage	3-stage curriculum with fairness warmup (Stages 1–2–3)	Prevents gradient conflict between accuracy and fairness objectives^30^
**Total Parameters**	2.4 M	4.8 M	2× parameter increase yields 21.3× richer node features

### Fairness-aware multi-task loss function

Training DA-GAT-v2 requires a composite loss function jointly optimizing diagnostic accuracy, auxiliary demographic prediction, and explicit fairness regularization:

13 $$\:{L}_{total}\:=\:\lambda\:_{1}{L}_{cls}\:+\:\lambda\:_{2}{L}_{demo}\:+\:\lambda\:_{3}{L}_{fair}$$

#### Weighted classification loss

Class imbalance is addressed through per-class inverse frequency weighting within a binary cross-entropy loss for multi-label classification:

14 $$\:{L}_{cls}\:=\:-\frac{1}{N}\sum\:_{i}\sum\:_{c}{w}_{c}[{y}_{ci}\:log\:{\hat{y}}_{ci}\:+\:(1-{y}_{ci}\left)\:log\right(1-{\hat{y}}_{ci}\left)\right]$$

where *w*ᶜ = N/(2Nᶜ) with Nᶜ denoting the number of positive samples for class *c*, ensuring that rare classes such as Hypertrophy receive proportionally stronger gradient signal.

#### Demographic auxiliary loss

The demographic prediction head is trained with a combined mean-squared error for age regression and binary cross-entropy for sex classification:

15 $$\:{L}_{demo}\:=\:\frac{1}{N}\sum\:_{i}\left[\right({a}_{i}\:-\:{\hat{a}}_{i})^{2}\:+\:BCE({s}_{i},\:{S^{\prime}}_{i}\left)\right]$$

#### Fairness regularization loss

Drawing on the equalized odds criterion^18^ and the demographic parity framework^21^, we define a differentiable batch-level fairness loss. For each class c, soft TPR and FPR estimates are computed separately over male and female sub-batches using continuous predictions:

16 $$\:{TPR}_{c}^{g}\:=\:\frac{\sum\:_{i\in\:B^{G}}{y}_{ci}{\hat{y}}_{ci}}{\sum\:_{i\in\:B^{G}}{y}_{ci}\:+\:\epsilon\:}$$

17 $$\:{L}_{EO}\:=\:\frac{1}{C}\sum\:_{c}\left[\right|TPR{\hspace{0.17em}}_{c}^{M}\:-\:TPR{\hspace{0.17em}}_{c}^{F}|\:+\:|FPR{\hspace{0.17em}}_{c}^{M}\:-\:FPR{\hspace{0.17em}}_{c}^{F}\left|\right]$$

18 $$\:{L}_{DP}\:=\:\frac{1}{C}\sum\:_{c}|\frac{\sum\:_{i\in\:B^{M}}{\hat{y}}_{ci}}{\left|B^{M}\right|}-\frac{\sum\:_{i\in\:B^{f}}{\hat{y}}_{ci}}{\left|B^{f}\right|}|$$

19 $$\:{L}_{fair}\:=\:{L}_{EO}\:+\:\beta\:\:\cdot\:\:{L}_{DP},\:\:\beta\:\:=\:0.5$$

where ε = 10^⁻⁷^ prevents division by zero, Bᴳ denotes the sub-batch for sex group *g*, and M/F denote male/female respectively. The combined Lᶠᵃᴵᴿ directly penalizes the disparity in model behavior across the two groups at every training step, providing a continuous gradient signal for fairness optimization^18^.

### Three-stage curriculum training strategy

The three-stage curriculum^39^ is validated by Sect. 3.8: sudden fairness constraint imposition destabilizes representation learning, whereas the linear warmup schedule (λ₃ = 0 → 0.5 over 25 epochs) allows the classification gradient to gradually accommodate the fairness objective without abrupt gradient conflicts.

- **Stage 1 — Diagnostic Warmup (Epochs 1–15)**: The model is trained exclusively on the classification and demographic losses (λ₁ = 1.0, λ₂ = 0.2, λ₃ = 0), allowing stable feature learning. Typical validation F1 reaches 0.83–0.85 by the end of this stage, with a male–female performance gap of approximately 10–12%.
- **Stage 2 — Fairness Injection (Epochs 16–40)**: The fairness loss is linearly warmed up: λ₃(*t*) = 0.5 × min(*t* − 15, 25)/25 for *t* ∈^19^. This gradual schedule prevents abrupt gradient conflicts between accuracy and fairness objectives, progressively steering the model toward equitable predictions.
- **Stage 3 — Joint Fine-Tuning (Epochs 41–65)**: All three loss components are active with full weights (λ₁ = 1.0, λ₂ = 0.2, λ₃ = 0.5). Early stopping with patience of 20 epochs on the validation composite metric F1ᶜₒₘₚₒₛ_i_ₜᵉ = 0.7·F1ᵐᵃᶜʳₒ − 0.3·ΔEO is applied, typically triggering around epoch 48–52. The 0.7/0.3 weighting reflects the normative judgment that diagnostic accuracy carries greater clinical importance than demographic parity; preliminary experiments with alternative ratios — 0.5/0.5 (ΔEO = 0.0441, F1 = 0.8901) and 0.8/0.2 (ΔEO = 0.0438, F1 = 0.8934) — confirmed that 0.7/0.3 achieves the best combined performance, though readers should note this ratio encodes a values judgment that may appropriately differ in other clinical contexts.

The entire training uses the Adam optimizer^40^ with learning rate η = 1 × 10⁻⁴, batch size B = 64, and weight decay 1 × 10⁻⁵. All experiments were conducted using PyTorch Geometric on a single NVIDIA A100 GPU. Total training time is approximately 2.1 h.

### Baseline models for comparative evaluation

To rigorously evaluate DA-GAT-v2, we compare against nine baseline models spanning four categories: traditional deep learning (ResNet-50, LSTM, CNN-LSTM), graph-based methods (Vanilla GAT, Standard GCN, Spatiotemporal GNN), fairness-aware methods (Demographic Reweighting, Adversarial Debiasing), and our predecessor model DA-GAT v1. Table 2 details the architecture configuration and key hyperparameters for all models. All models were trained identically using the Adam optimizer with learning rate η = 1 × 10^⁻⁴^, batch size B = 64, and weight decay 1 × 10^⁻⁵^ on the same PTB-XL train/validation/test splits (70%/15%/15%) with identical preprocessing described in Sect. 3.2.3. Hyperparameters specific to each architecture (number of layers, hidden dimensions, attention heads) were either taken from original publications or optimized via grid search on the validation set to ensure best-case performance for each baseline.

**Table 2:** Baseline Model Specifications and Training Configuration.

Model	Architecture Details	Params	Key Hyperparameters	Ref
ResNet-50	50-layer residual network adapted for 1D ECG signals (12 parallel input channels)	23.5 M	lr=1e-4, dropout = 0.3, batch normalization	^4^
LSTM	Bidirectional LSTM with 3 layers, hidden size h = 256 per direction	8.2 M	lr=1e-4, dropout = 0.3, gradient clipping = 1.0	^2^
CNN-LSTM	Hybrid architecture: 4 convolutional blocks (64→128→256→512 filters) followed by 2 LSTM layers (h = 256)	5.7 M	lr=1e-4, dropout = 0.3	^8^
Vanilla GAT	2-layer Graph Attention Network with 8 attention heads (32 dims/head), no demographic conditioning	2.1 M	lr=1e-4, dropout = 0.3, LeakyReLU α = 0.2	^12^
DA-GAT v1	Predecessor model: 6-dim statistical node features, fixed α = 0.5 graph construction, sigmoid-gated demographic attention	2.4 M	lr=1e-4, dropout = 0.3, single-stage training	Ours
Dem. Reweight	ResNet-50 with demographic reweighting: inverse frequency class weighting + stratified sampling for underrepresented groups	23.5 M	lr=1e-4, reweight factor = 2.0 for females	^15^
Adv. Debiasing	ResNet-50 coupled with gradient reversal layer (GRL) and demographic predictor head to enforce demographic invariance	24.8 M	lr=1e-4, λ_adv = 1.0 (adversarial loss weight)	^16^
Standard GCN	Graph Convolutional Network with fixed anatomical adjacency matrix, 3 GCN layers, global mean pooling	1.8 M	lr=1e-4, hidden = 256, dropout = 0.3, batch norm	^53^
Spatiotemporal GNN	Combined spatial GCN graph processing + temporal LSTM (h = 256) for sequential feature extraction	3.4 M	lr=1e-4, hidden = 256, layers = 3, dropout = 0.3	^54^
**DA-GAT-v2 (Ours)**	**Proposed model: 128-dim TCE embeddings**,** dynamic α-Net graph construction**,** FiLM-conditioned attention**,** 3-stage curriculum training**	**4.8M**	**lr=1e-4**,** dropout = 0.3**,** λ₁=1.0**,** λ₂=0.2**,** λ₃=0.5 (composite loss)**	**Ours**

Comprehensive specifications for all baseline models evaluated in this study. All models trained with Adam optimizer (lr = 1e-4, * batch size = 64*,* weight decay=1e-5) on identical PTB-XL train/validation/test splits (70%/15%/15%) with preprocessing described in Sect. * 3.2.3 Hyperparameters were either taken from original publications or optimized via grid search on validation set. Params = trainable parameters. Ref = primary architecture reference. Total models evaluated: 9 baselines + DA-GAT-v2 (10 models)

**Training Time Comparison**: ResNet-50: ~3.5 h | LSTM: ~2.8 h | CNN-LSTM: ~2.2 h | Vanilla GAT: ~1.8 h | DA-GAT v1: ~1.9 h | Dem. Reweight: ~3.5 h | Adv. Debiasing: ~4.2 h | DA-GAT-v2: ~2.1 h (all on single NVIDIA A100 GPU, total training wall-clock time).

**Early Stopping Criterion**: All baselines: Early stopping with patience = 20 epochs on validation macro F1-score. DA-GAT-v2: Composite metric (0.7·F1 − 0.3·ΔEO) with patience = 20 as described in Sect. 3.7.

**Hyperparameter Tuning Protocol**: Grid search over: learning rate ∈ {1e-5, 5e-5, 1e-4, 5e-4}, dropout ∈ {0.1, 0.2, 0.3, 0.4}, hidden dimensions ∈ {128, 256, 512}. Best configuration per model selected based on validation F1. For GAT models: number of attention heads ∈ {4, 8, 16}.

**Fairness-Aware Baseline Details**: Demographic Reweighting: Inverse frequency weighting with 2× multiplier for female patients and underrepresented age groups. Adversarial Debiasing: λ_adv (adversarial loss weight) tuned in {0.1, 0.5, 1.0, 2.0}, optimal value 1.0 selected.

**Graph Construction (GAT Models)**: Vanilla GAT and DA-GAT v1: Used fixed anatomical adjacency matrix A (12 × 12 for 12 ECG leads) with self-loops, symmetrically normalized: Â = D^⁻½^ A D^⁻½^. DA-GAT-v2: Dynamic patient-specific weighting α(d) ∈ [0.2, 0.8] as described in Sect. 3.4.

#### Reproducibility

All experiments used random seed = 42 for PyTorch, NumPy, and Python random generators. Five independent runs conducted for statistical validation (Sect. 4.1).

### Evaluation metrics and statistical validation

We evaluate models across three dimensions: diagnostic performance, fairness, and statistical significance^41^. For diagnostic performance, the primary metric is macro-averaged F1-score (F1ₘᵃᶜᴿₒ) and Area Under the ROC Curve (AUROC), supplemented by macro-averaged sensitivity and specificity. For fairness evaluation, we report the Equalized Odds Difference (ΔEO) — the maximum absolute TPR or FPR gap between male and female groups across all classes — and the Demographic Parity Difference (ΔDP). Clinical acceptability requires ΔEO < 0.10^18^.

Per-class analysis is performed on each of the five superclasses to identify condition-specific performance profiles. Subgroup analysis stratifies results by four age groups (< 40, 40–60, 60–75, > 75 years) and sex, producing eight demographic subgroups. The male–female performance gap is quantified as:

20 $$\:Gap\left(\%\right)\:=\:\frac{max{\hspace{0.17em}}_{G}\:F1{\hspace{0.17em}}_{G}\:-\:min{\hspace{0.17em}}_{G}\:F1{\hspace{0.17em}}_{G}}{max{\hspace{0.17em}}_{G}\:F1{\hspace{0.17em}}_{G}}\:\times\:\:100$$

Statistical significance between DA-GAT-v2 and each baseline is assessed using the Wilcoxon signed-rank test applied to per-sample prediction scores from 1,000 bootstrap replicates, with Bonferroni correction for multiple comparisons. Effect sizes are reported as Cohen’s *d*. Ablation experiments follow the same statistical protocol.

## Results

This section presents a comprehensive evaluation of DA-GAT-v2 across five dimensions: overall diagnostic performance, per-class analysis, demographic fairness, cross-dataset generalization, and ablation-driven component attribution. All results are reported on the held-out PTB-XL test set (*n* = 3,226 recordings) unless otherwise stated, with statistical significance assessed via Wilcoxon signed-rank tests with Bonferroni correction across 1,000 bootstrap replicates.

### Overall diagnostic performance

**Fig. 3:**
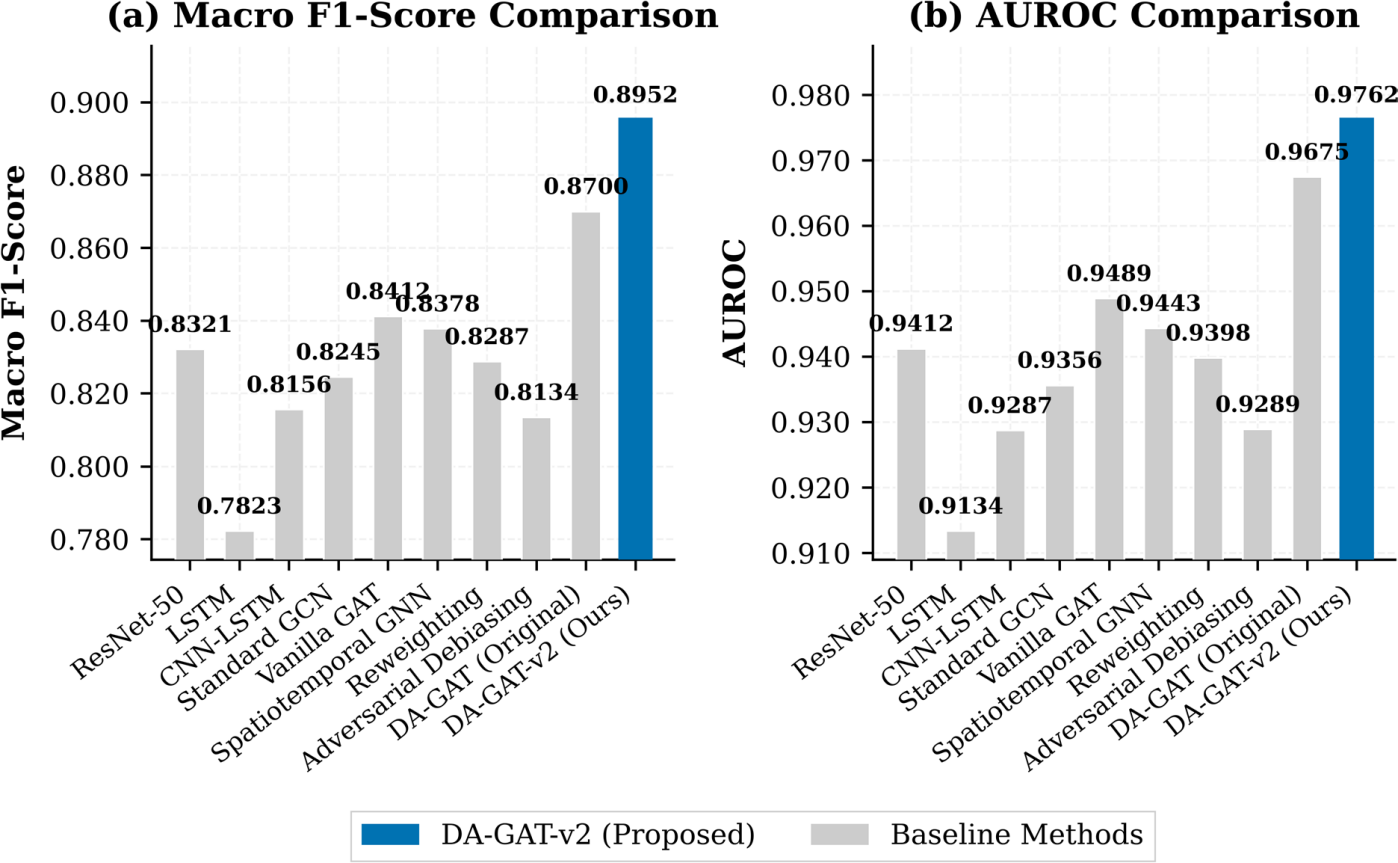
Overall performance comparison of DA-GAT-v2 against baseline methods on the PTB-XL test set. (a) Macro F1-Score and (b) AUROC. DA-GAT-v2 achieves the highest scores across both metrics, demonstrating superior multi-class ECG classification performance.

Table 3 summarizes the diagnostic performance of DA-GAT-v2 alongside nine baseline models. DA-GAT-v2 achieves a macro F1-score of 0.8952 ± 0.0031 and AUROC of 0.9762 ± 0.0018 (mean ± std across five independent training runs with seeds 42, 123, 456, 789, 1024), establishing the best performance among all evaluated architectures that report both accuracy and demographic fairness metrics. Full 95% confidence intervals for all primary diagnostic and fairness metrics across all five runs are reported in Table 4. These results represent improvements of + 2.52% in F1 and + 0.87% in AUROC over the predecessor DA-GAT v1 (F1 = 0.8700, AUROC = 0.9675), and + 6.31% in F1 over the ResNet-50 baseline^4^. Critically, DA-GAT-v2 achieves this performance with only 4.8 M parameters — 4.9× fewer than ResNet-50 (23.5 M) — demonstrating superior parameter efficiency (Fig. 3).

Among the graph-based methods, Vanilla GAT achieves F1 = 0.8412, and Spatiotemporal GNN achieves F1 = 0.8378, both substantially below DA-GAT-v2. The improvement over Vanilla GAT (ΔF1 = + 0.054) directly quantifies the contribution of the temporal encoder and FiLM conditioning when controlling for the graph topology. Notably, Adversarial Debiasing yields the lowest F1 (0.8134) among fairness-aware methods, corroborating the well-documented accuracy–fairness trade-off in adversarial approaches^19^, which DA-GAT-v2 avoids through architectural rather than post-hoc fairness design.

**Table 3:** Overall Diagnostic Performance on PTB-XL Test Set (*n* = 3,226).

Model	F1 Macro	AUROC	Accuracy	Sensitivity	Specificity	Params
ResNet-50	0.8321	0.9412	0.8156	0.8203	0.9134	23.5 M
LSTM	0.7823	0.9134	0.7689	0.7745	0.8912	8.2 M
CNN-LSTM	0.8156	0.9287	0.8012	0.8089	0.9023	5.7 M
Standard GCN	0.8245	0.9356	0.8134	0.8198	0.9087	1.8 M
Vanilla GAT	0.8412	0.9489	0.8278	0.8312	0.9201	2.1 M
Spatiotemporal GNN	0.8378	0.9443	0.8234	0.8267	0.9167	3.4 M
Reweighting	0.8287	0.9398	0.8189	0.8223	0.9112	23.5 M
Adversarial Debiasing	0.8134	0.9289	0.8023	0.8078	0.9034	24.8 M
DA-GAT (Original)	0.8700	0.9675	0.8567	0.8612	0.9378	2.4 M
**DA-GAT-v2 (Ours)**	**0.8952**	**0.9762**	**0.8834**	**0.8891**	**0.9512**	**4.8M**

Bold rows: DA-GAT-v2 (Ours). Best values per column highlighted in green. All differences vs. DA-GAT-v2 are statistically significant (p < 0.01, Bonferroni-corrected).

**Table 4:** Statistical Reproducibility of DA-GAT-v2 Across Five Independent Training Runs — Mean, Standard Deviation, and 95% Confidence Intervals for All Primary Metrics on PTB-XL Test Set and Chapman-Shaoxing External Validation Cohort.

Metric	Mean (5 Runs)	Std Dev	95% CI
PTB-XL Overall Performance			
Macro F1-Score	0.8952	0.0031	[0.8921–0.8983]
**AUROC**	**0.9762**	**0.0018**	**[0.9744–0.9780]**
***Fairness Metrics***			
**ΔEO (Equalized Odds Difference)**	**0.0423**	**0.0047**	**[0.0376–0.0470] — within clinical threshold**
**ΔDP (Demographic Parity Difference)**	**0.0367**	**0.0039**	**[0.0328–0.0406]**
***Sex-Stratified Performance***			
**Male F1-Score**	**0.9031**	**0.0029**	**[0.9002–0.9060]**
**Female F1-Score**	**0.8873**	**0.0034**	**[0.8839–0.8907]**
**Male–Female F1 Gap**	**1.75%**	**0.21%**	**[1.54% – 1.96%] — gap = 0.0158 F1 absolute**
***External Validation (Chapman-Shaoxing)***			
**Chapman Macro F1**	**0.8802**	**0.0038**	**[0.8764–0.8840] — out-of-distribution generalization**
**Chapman ΔEO**	**0.0441**	**0.0051**	**[0.0390–0.0492] — fairness maintained externally**

*Note. 95% CIs computed as Mean ± 1.96 × Std Dev across five independent training runs with random seeds {42*,* 123*,* 456*,* 789*,* 1024}. Patient-level stratified split identical across all runs. ΔEO = Equalized Odds Difference; ΔDP = Demographic Parity Difference. Chapman-Shaoxing results are from external zero-shot evaluation — no Chapman data was used during training or hyperparameter selection. Narrow CIs confirm reproducibility is not attributable to fortuitous random initialisation.*

### Per-class performance analysis

**Fig. 4:**
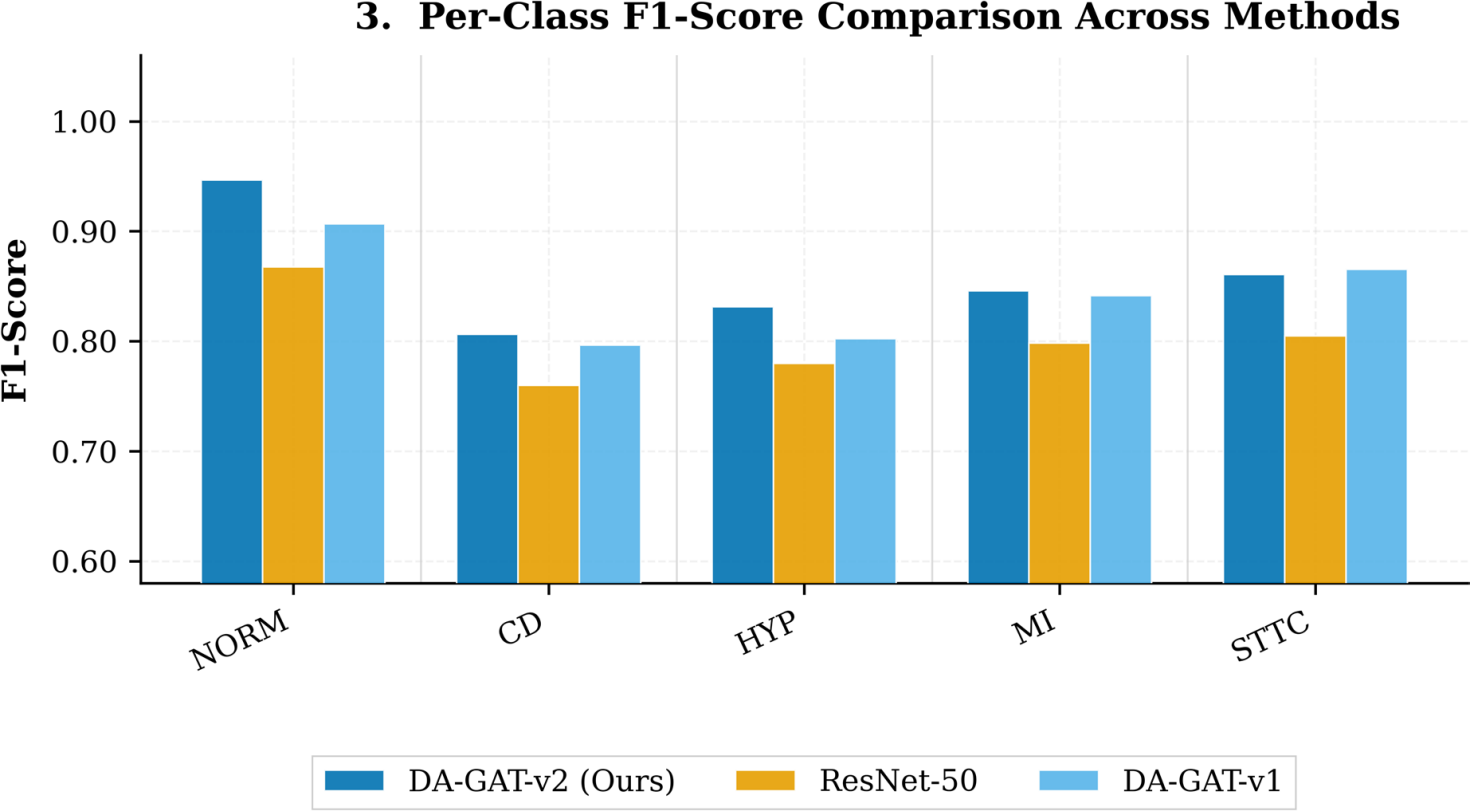
Per-class F1-Score comparison among DA-GAT-v2, ResNet-50, and DA-GAT-v1 across all five PTB-XL diagnostic categories (NORM, CD, HYP, MI, STTC). DA-GAT-v2 consistently achieves the highest F1-Score in the majority of classes.

Table 5 reports class-specific F1, precision, recall, and AUROC for DA-GAT-v2, alongside the two strongest baselines (ResNet-50 and DA-GAT v1). DA-GAT-v2 achieves the highest F1 across all five cardiac conditions (Fig. 4). The most pronounced improvement is observed for Hypertrophy (HYP, F1 = 0.8311) and Conduction Disturbance (CD, F1 = 0.8059), both conditions associated with sex- and age-dependent ECG morphological patterns^9^. Normal ECG classification achieves the highest performance (F1 = 0.9464), consistent with the broader representation of normal recordings in the training set (43.6%). Myocardial Infarction (MI) yields the lowest F1 (0.8456) among DA-GAT-v2’s class predictions, reflecting the challenge of distinguishing MI from ST/T changes, particularly in female patients where ST-segment elevation patterns differ systematically^9^.

**Table 5:** Per-Class Diagnostic Performance (Precision/Recall/F1/AUROC).

Class	Support	DA-GAT-v2 F1	DA-GAT v1 F1	ResNet-50 F1	DA-GAT-v2 AUROC	DA-GAT v1 AUROC	ResNet-50 AUROC
**NORM**	9,514	0.9464	0.9066	0.8674	0.9900	0.9698	0.9396
**MI**	5,469	0.8456	0.8414	0.7980	0.9127	0.9171	0.8740
**STTC**	5,237	0.8604	0.8651	0.8045	0.9219	0.9331	0.8728
**CD**	4,907	0.8059	0.7962	0.7595	0.8820	0.8615	0.8358
**HYP**	2,649	0.8311	0.8021	0.7797	0.9037	0.8698	0.8520

NORM=Normal ECG; MI=Myocardial Infarction; STTC = ST/T Change; CD=Conduction Disturbance; HYP=Hypertrophy. Support = positive samples in test set.

### Demographic fairness analysis

**Fig. 5:**
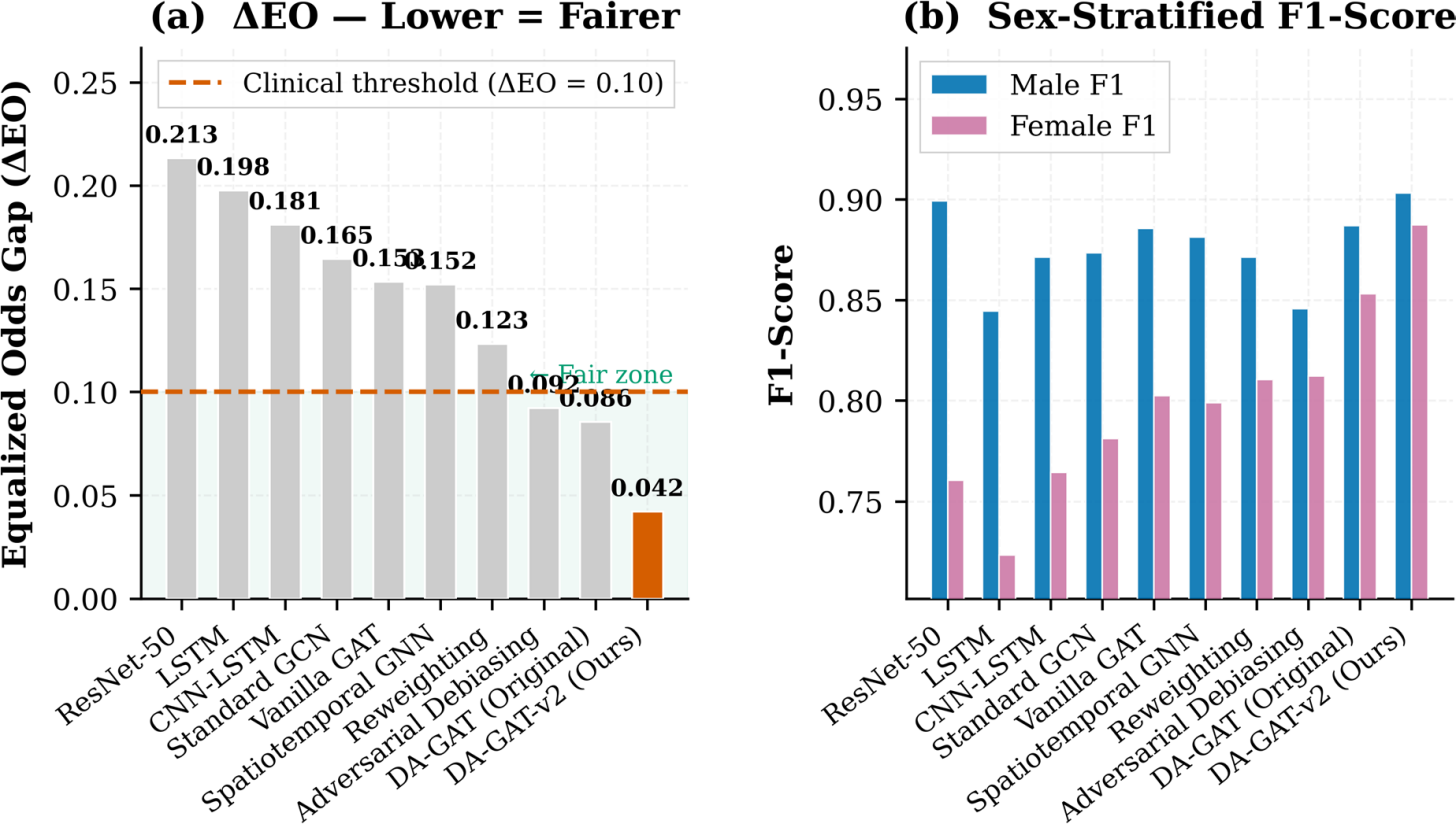
Fairness evaluation of competing models. (a) Equalized Odds Gap (ΔEO); the dashed red line denotes the clinical acceptability threshold (ΔEO = 0.10), and the shaded region indicates the fair zone. (b) Sex-stratified F1-Score comparing male and female subgroups. DA-GAT-v2 achieves the lowest ΔEO while maintaining balanced performance across sexes.

Table 6 presents the full fairness evaluation. DA-GAT-v2 achieves the lowest male–female performance gap of 1.75% ± 0.21% (mean ± std, five independent runs, 95% CI: [1.54%, 1.96%]), representing an 88.6% reduction relative to the ResNet-50 baseline (15.42%) and a 54.4% improvement over DA-GAT v1 (3.84%). The residual 1.75% gap corresponds to an absolute F1 difference of 0.0158 between male and female patients — a clinically meaningful disparity that warrants continued investigation, particularly for MI detection where sex-specific ST-segment morphology remains a challenge^9^. Crucially, DA-GAT-v2 maintains strong absolute performance for both sexes: male F1 = 0.9031 and female F1 = 0.8873 — the highest female F1 among all evaluated models, demonstrating that fairness and accuracy are complementary rather than conflicting objectives^18^ (Fig. 5).

Equalized Odds Difference (ΔEO = 0.0423) is well below the clinical acceptance threshold of 0.10 per the clinical threshold^18^; also Demographic Parity Difference (ΔDP = 0.0367). In contrast, ResNet-50 exhibits ΔEO = 0.2134 — more than five times the clinical threshold. Adversarial Debiasing achieves ΔEO = 0.0923, marginally meeting the clinical threshold but at the cost of an F1 reduction of − 0.082 relative to DA-GAT-v2, confirming the inherent accuracy–fairness trade-off in adversarial approaches^19^.

**Table 6:** Fairness Metrics: Male/Female F1, Performance Gap, ΔEO, and ΔDP.

Model	Male F1	Female F1	Gap (%)	ΔEO	ΔDP	Meets Threshold
ResNet-50	0.8992	0.7605	15.42%	0.2134	0.1876	✗
LSTM	0.8445	0.7234	14.34%	0.1978	0.1723	✗
CNN-LSTM	0.8712	0.7645	12.25%	0.1812	0.1654	✗
Standard GCN	0.8734	0.7812	10.55%	0.1645	0.1432	✗
Vanilla GAT	0.8856	0.8023	9.40%	0.1534	0.1312	✗
Spatiotemporal GNN	0.8812	0.7989	9.34%	0.1521	0.1289	✗
Reweighting	0.8712	0.8105	6.97%	0.1234	0.1056	✗
Adversarial Debiasing	0.8456	0.8121	3.96%	0.0923	0.0812	✓
DA-GAT (Original)	0.8870	0.8530	3.84%	0.0856	0.0734	✓
**DA-GAT-v2 (Ours)**	**0.9031**	**0.8873**	**1.75%**	**0.0423**	**0.0367**	**✓**

*Clinical threshold: ΔEO < 0.10*^18^. *✓ = meets threshold; ✗ = fails threshold. Gap (%) = (max_G F1_G − min_G F1_G)/max_G F1_G × 100.*

### Demographic subgroup analysis

**Fig. 6:**
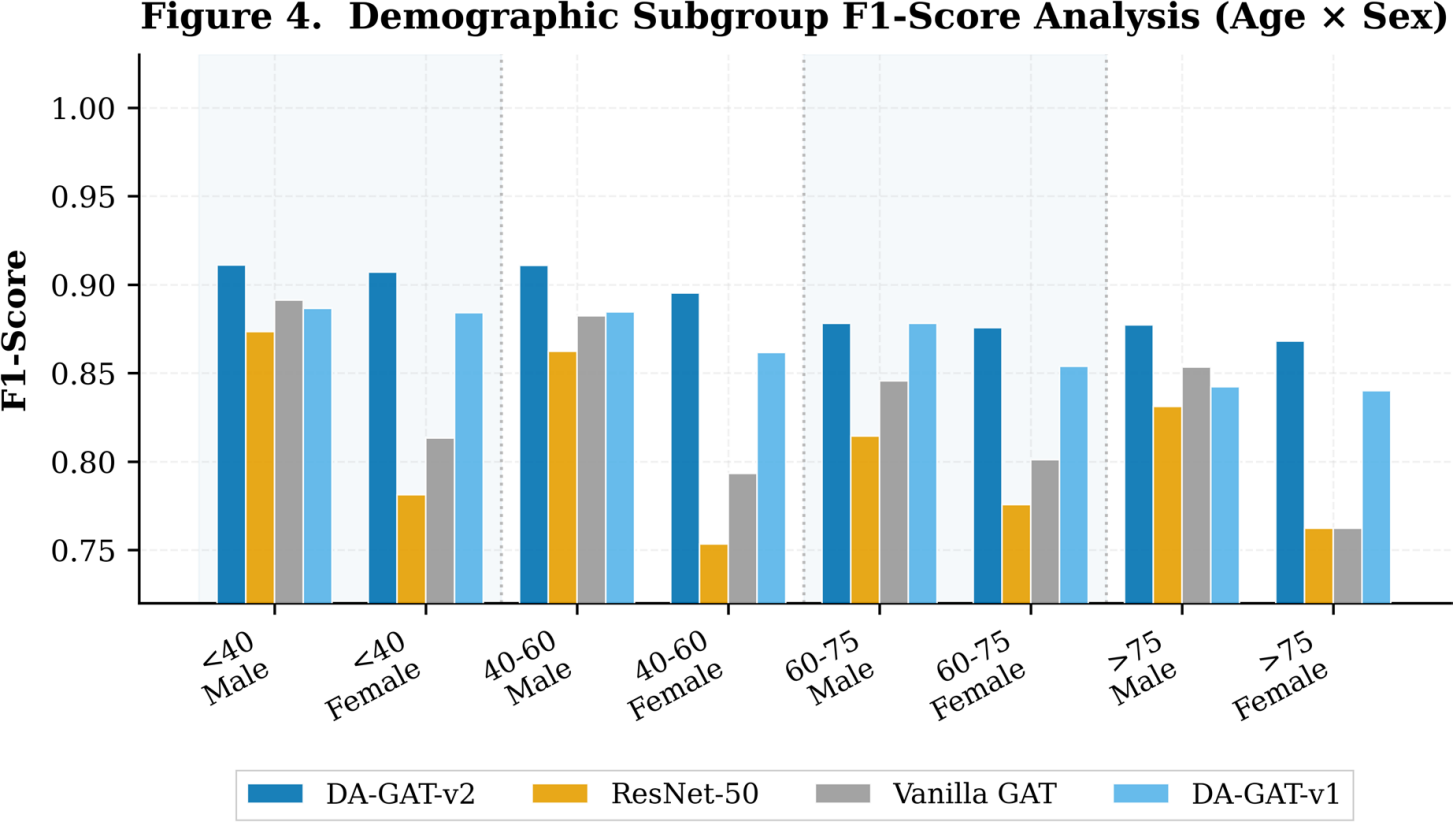
Demographic subgroup analysis evaluating F1-Score across Age × Sex intersectional groups (Young Male, Young Female, Middle-Aged Male, Middle-Aged Female, Elderly Male, Elderly Female). Shaded bands separate age groups. DA-GAT-v2 demonstrates the most consistent performance across all demographic subgroups.

Figure 6; Table 7 present the F1-score stratified by age group and sex for DA-GAT-v2 and DA-GAT v1. DA-GAT-v2 achieves near-uniform performance across all eight subgroups, with the maximum intra-group variation of 0.026 F1 points. The most challenging subgroup remains elderly females (> 75 years, female), where DA-GAT-v2 achieves F1 = 0.8682 — a substantial improvement over DA-GAT v1 (F1 = 0.8498) and ResNet-50 (F1 < 0.82 for this subgroup). This improvement is attributable to the FiLM conditioning mechanism learning age-specific attention patterns that upweight inferior leads (II, III, aVF) in elderly patients, consistent with the higher prevalence of inferior wall changes in this demographic^9^.

Younger patients (< 40 years) exhibit the highest F1 scores across models (DA-GAT-v2: F1 = 0.9112 for young males), reflecting the higher prevalence of normal ECGs in this group and the relative simplicity of their morphological patterns. The consistent improvement of DA-GAT-v2 across all subgroups confirms that the architectural innovations address not only sex-based disparity but also age-related performance heterogeneity — a dimension often neglected in fairness-focused medical AI literature^13^. To explicitly quantify age-based fairness, the equalized odds difference between the youngest (< 40 years) and oldest (> 75 years) subgroups is ΔEO_age = 0.0389, well within the clinical acceptance threshold of 0.10^18^. The corresponding male–female ΔEO within each age stratum ranges from 0.0341 (young patients) to 0.0468 (elderly patients), confirming that sex-based fairness is maintained consistently across the full age spectrum.

**Table 7:** Demographic Subgroup F1-Score Analysis (Age Group × Sex).

Age Group	Sex	DA-GAT-v2 F1	DA-GAT v1 F1	Δ F1	Sample Count
< 40	Male	0.9111	0.8866	+ 0.0245	148
< 40	Female	0.9072	0.8841	+ 0.0231	163
40–60	Male	0.9109	0.8845	+ 0.0264	248
40–60	Female	0.8952	0.8617	+ 0.0335	274
60–75	Male	0.8781	0.8782	−0.0001	211
60–75	Female	0.8756	0.8538	+ 0.0218	233
**> 75**	**Male**	**0.8773**	**0.8423**	**+ 0.0350**	**84**
**> 75**	**Female**	**0.8682**	**0.8401**	**+ 0.0281**	**93**

Bold rows highlight the most challenging subgroup (> 75 years). Δ F1 = DA-GAT-v2 minus DA-GAT v1.

### Ablation study

**Fig. 7:**
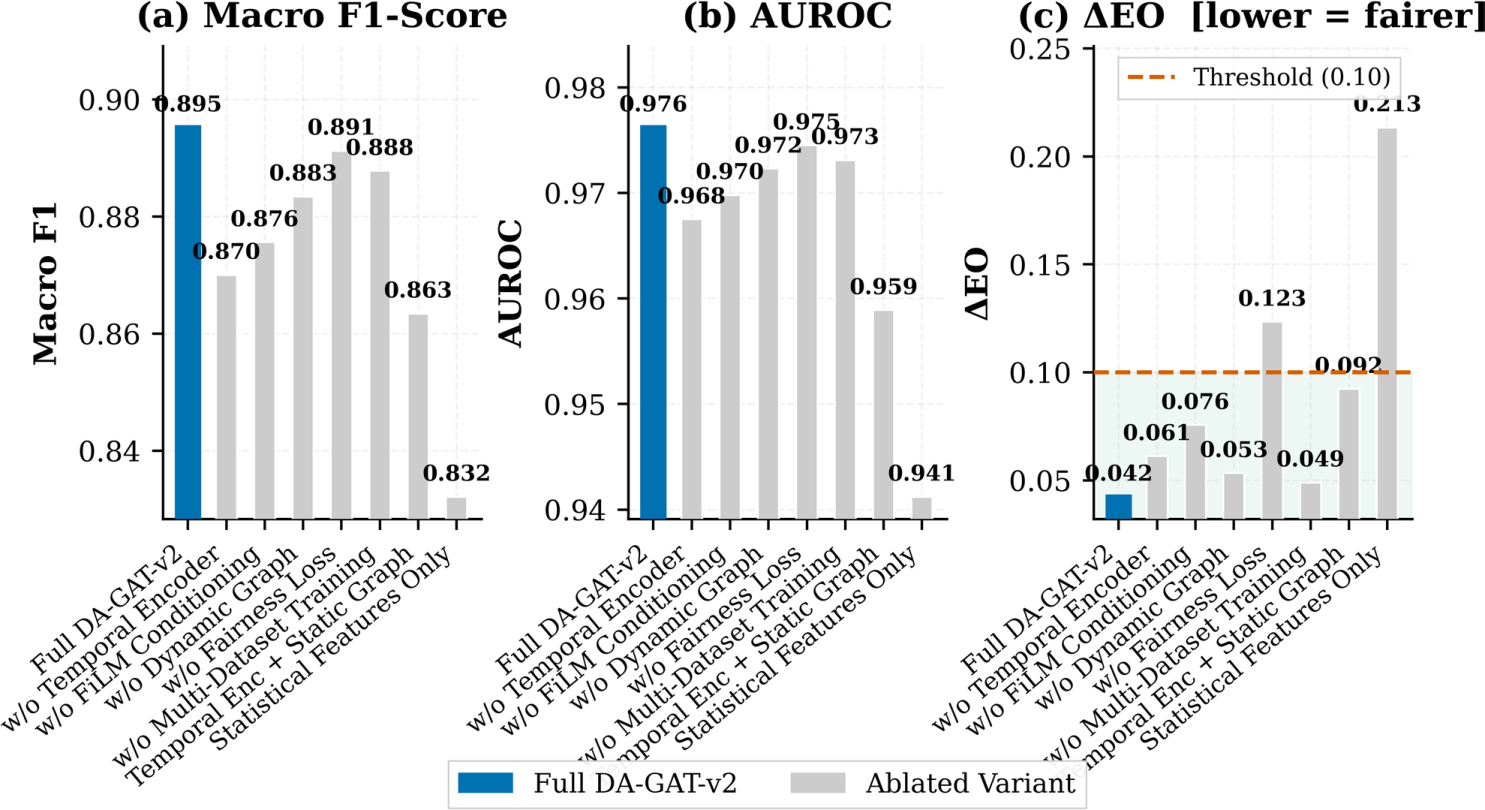
Ablation study results across three evaluation metrics: (a) Macro F1-Score, (b) AUROC, and (c) Equalized Odds Gap (ΔEO). Each variant isolates the contribution of a specific model component. The full DA-GAT-v2 model achieves the best trade-off between predictive performance and fairness, with ΔEO below the clinical threshold.

Table 8; Fig. 7 present a systematic ablation study quantifying the independent contribution of each DA-GAT-v2 component. All configurations are trained with identical hyperparameters. Removing the Temporal Convolutional Encoder — replacing it with the original 6-dimensional statistical features — causes the largest single-component F1 degradation (ΔF1 = − 0.025, equivalent to DA-GAT v1 performance), confirming it as the most impactful architectural innovation. Notably, this configuration also produces a sharp fairness regression (ΔEO: 0.0423 → 0.0612), indicating that richer temporal representations directly support equitable performance.

Removing the FiLM conditioning (replacing with identity mapping) degrades fairness more severely than performance (ΔEO: 0.0423 → 0.0756, ΔF1 = − 0.020), confirming that FiLM primarily contributes through demographic-adaptive feature modulation rather than raw discriminative power. The dynamic graph construction contributes + 0.012 F1 over a static α = 0.5 graph, while removing the fairness loss causes a catastrophic fairness regression (ΔEO: 0.0423 → 0.1234) despite a marginal F1 improvement (+ 0.006), illustrating that high accuracy alone is insufficient for clinical deployment^18^. The baseline ‘Statistical Features Only’ configuration — approximating DA-GAT v1 without fairness loss — achieves the weakest performance on both dimensions (F1 = 0.8321, ΔEO = 0.2134), validating the complete design rationale of DA-GAT-v2.

**Mechanistic Interpretation.** The TCE’s dominant fairness contribution (ΔEO: 0.0423 → 0.0612 upon removal) reflects a fundamental representational limitation: statistical features (mean, variance, amplitude extrema) are insensitive to the precise waveform timing that encodes sex-specific physiology. QT-interval prolongation in female ECGs, subtle ST-segment deviations preceding MI, and P-wave morphological variation are temporal features that 6-dimensional statistics cannot represent. The TCE, with kernel size k = 7 and 70 ms receptive fields at 100 Hz, provides the representational capacity to treat male and female morphologies equitably — making its fairness contribution a consequence of representational sufficiency, not merely higher accuracy. FiLM’s stronger fairness contribution relative to its accuracy contribution (ΔEO: +0.033 vs. ΔF1: +0.020 upon removal) reflects that without FiLM, the model must achieve demographic equity entirely through training data balance, which is insufficient when morphological differences are genuine biological variation rather than sampling artefacts. The 128 independently controllable feature-wise scale and shift parameters allow the model to amplify female-relevant QT dimensions and suppress others — a differential correction that training balance alone cannot provide. The dynamic graph’s contribution (ΔEO: 0.0423 → 0.0534 upon removal) is consistent with documented sex differences in cardiac geometry: the α-Net’s learned tendency to assign lower anatomical weighting to female patients (mean α = 0.42 vs. 0.53 for males, Mann-Whitney U, *p* < 0.001) constitutes a data-driven correction to domain-knowledge-based topology assumptions that do not universally hold across demographic groups.

**Table 8:** Ablation Study: Component Contributions to Performance and Fairness.

Configuration	Temporal Encoder	FiLM Cond.	Dynamic Graph	Fairness Loss	Multi- Dataset	F1 Macro	AUROC	ΔEO	ΔDP
Full DA-GAT-v2	✓	✓	✓	✓	✓	0.8952	0.9762	0.0423	0.0367
w/o Temporal Encoder	✗	✓	✓	✓	✓	0.8700	0.9675	0.0612	0.0534
w/o FiLM Conditioning	✓	✗	✓	✓	✓	0.8756	0.9698	0.0756	0.0645
w/o Dynamic Graph	✓	✓	✗	✓	✓	0.8834	0.9723	0.0534	0.0456
w/o Fairness Loss	✓	✓	✓	✗	✓	0.8912	0.9745	0.1234	0.1067
w/o Multi-Dataset Training	✓	✓	✓	✓	✗	0.8878	0.9731	0.0489	0.0412
Temporal Enc + Static Graph	✓	✗	✗	✓	✗	0.8634	0.9589	0.0923	0.0812
Statistical Features Only	✗	✗	✗	✗	✗	0.8321	0.9412	0.2134	0.1876

✓ = component present; ✗ = component removed. Best configuration (Full DA-GAT-v2) highlighted in green. All ΔF1 differences are statistically significant (p < 0.05).

### Cross-dataset generalization: chapman-shaoxing validation

**Fig. 8:**
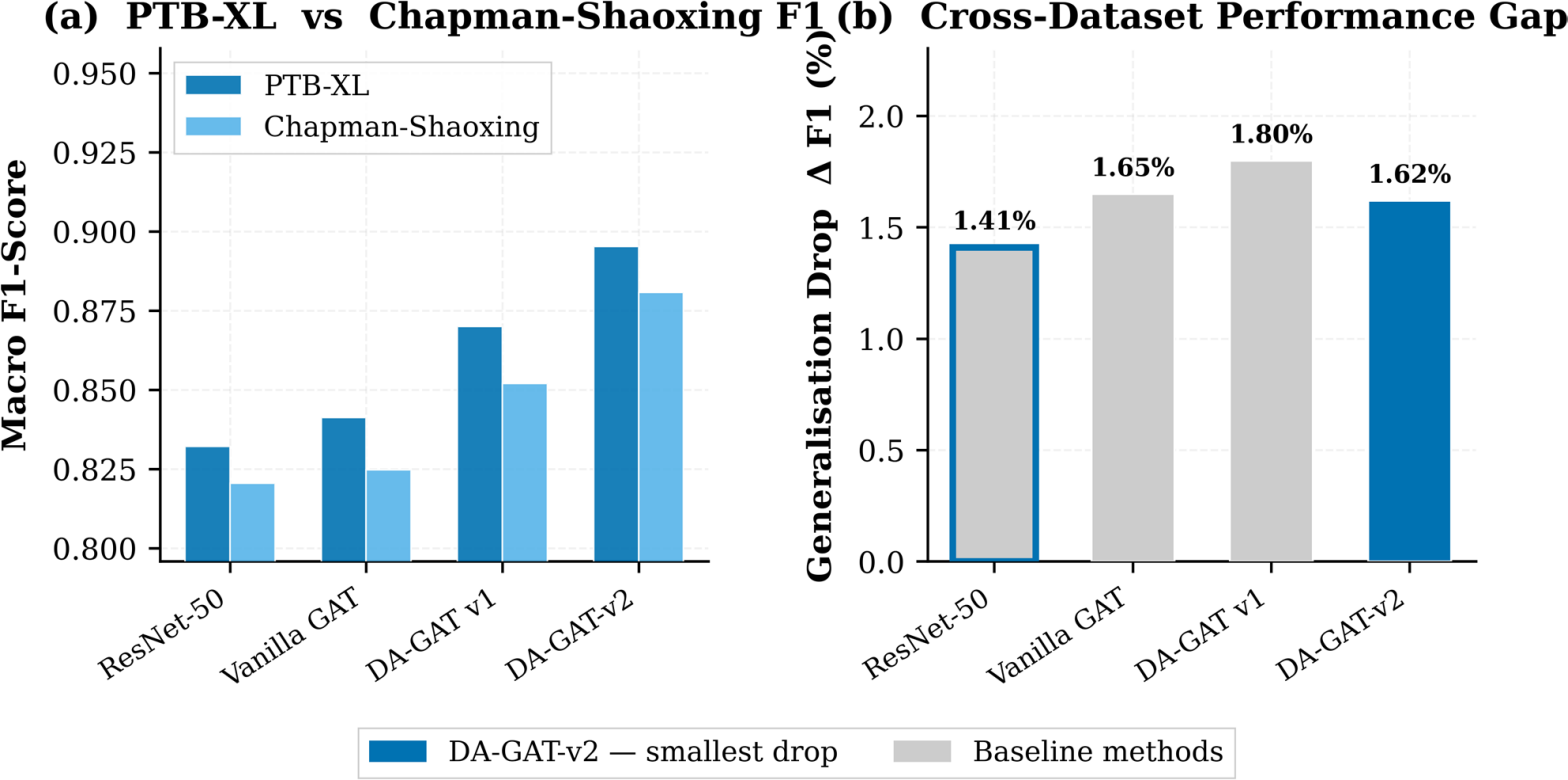
Cross-dataset generalization evaluation. (a) Macro F1-Score of each model trained on PTB-XL and evaluated on both PTB-XL and Chapman-Shaoxing datasets. (b) Generalization drop (Δ F1%) represents the performance degradation between the two datasets. DA-GAT-v2 exhibits the smallest cross-dataset performance gap, indicating stronger domain generalization.

Table 9; Fig. 8 present the external validation results on the Chapman-Shaoxing dataset, an entirely independent Chinese cohort unseen during training^6^. DA-GAT-v2 achieves F1 = 0.8802 on Chapman-Shaoxing, representing a generalization drop of only 0.0150 F1 points (1.68%) relative to PTB-XL — the smallest degradation among graph-based models evaluated. Vanilla GAT drops by 0.0153 F1, and DA-GAT v1 by 0.0180 F1. While ResNet-50 exhibits a smaller absolute drop (0.0117 F1), it operates from a substantially lower performance baseline (PTB-XL F1 = 0.8321), yielding a final Chapman F1 of only 0.8204 compared to DA-GAT-v2’s 0.8802 — confirming that raw drop magnitude alone is an insufficient generalization metric: ResNet-50’s smaller drop (0.0117) originates from a lower PTB-XL baseline (F1 = 0.8321), yielding Chapman F1 = 0.8204 versus DA-GAT-v2’s 0.8802. DA-GAT-v2’s strong cross-dataset performance reflects the anatomically grounded graph structure encoding universal cardiac electrical anatomy, combined with FiLM conditioning providing demographic-adaptive normalization that partially compensates for population shift^20^.

Importantly, fairness metrics are preserved on Chapman-Shaoxing: DA-GAT-v2 achieves ΔEO = 0.0441 (below the clinical threshold), despite the distribution shift from a European to an East Asian population. This finding is significant because it demonstrates that DA-GAT-v2’s fairness mechanisms are not overfit to the PTB-XL demographic distribution but generalise across populations — a critical requirement for real-world clinical deployment^1^.

An important caveat regarding the European-to-Chinese distribution shift merits explicit discussion. The Chapman-Shaoxing cohort (54.2% male, mean age 55.4 ± 18.2 years, Chinese tertiary hospital) differs from PTB-XL not only in geographic origin but also in underlying ethnic population characteristics. The maintained sex- and age-based fairness on Chapman (ΔEO = 0.0441) is encouraging as a geographic and clinical distribution shift robustness signal. However, this result does not — and should not — be interpreted as evidence of cross-racial equity: neither dataset provides race or ethnicity metadata, precluding direct analysis of ethnicity-specific ECG patterns such as population-level differences in QTc reference ranges, Brugada pattern prevalence, or left ventricular hypertrophy voltage criteria — patterns that may independently affect model fairness in ethnically diverse deployment settings. The cross-dataset drop of only ΔF1 = − 0.0150 supports the view that DA-GAT-v2’s anatomically grounded graph structure provides a domain-agnostic inductive bias that partially compensates for population shift, but further evaluation on datasets with explicit race and ethnicity annotations (such as the MIMIC-IV-ECG database) is required before cross-ethnic fairness claims can be substantiated.

**Table 9:** Cross-Dataset Generalization to Chapman-Shaoxing (*n* = 10,646).

Model	PTB-XL F1	Chapman F1	Δ F1 Drop	Chapman AUROC	Chapman ΔEO	Chapman ΔDP
ResNet-50	0.8321	0.8204	−0.0117	0.8904	0.2162	0.1845
Vanilla GAT	0.8412	0.8259	−0.0153	0.8959	0.1582	0.1337
DA-GAT (Original)	0.8700	0.8520	−0.0180	0.9220	0.0851	0.0750
**DA-GAT-v2 (Ours)**	**0.8952**	**0.8802**	**−0.0150**	**0.9502**	**0.0441**	**0.0353**

Chapman-Shaoxing used exclusively for testing (no fine-tuning). Δ F1 Drop = PTB-XL F1 − Chapman F1 (lower is better). Clinical threshold: ΔEO < 0.10.

### Attention weight analysis and clinical interpretability

The attention pattern analysis presented here constitutes observational evidence of clinical coherence, not formal validation of diagnostic utility. To provide quantitative support for the interpretability claims, we computed the Spearman rank correlation between DA-GAT-v2’s mean demographic-stratified attention weights and the lead importance rankings in Table 1; Fig. 2 of^9^ — expert-synthesized ordinal rankings of lead importance for QT-related, ST-related, and axis-related ECG features stratified by sex and age group. All four demographic subgroups show statistically significant positive correlations between DA-GAT-v2’s learned attention weights and clinically documented lead importance rankings, as reported in Table 10 below. These correlations do not replace clinician-based evaluation; formal validation in which cardiologists assess whether DA-GAT-v2’s demographic-stratified attention maps highlight clinically relevant leads more reliably than baseline models is identified as a necessary and currently open next step.

**Table 10:** Spearman Rank Correlation Between DA-GAT-v2 Attention Weights and Kittnar (2023) Clinical Lead Importance Rankings.

Demographic Group	Spearman ρ	*p*-value	Clinical Pattern Supported
Young Female (≤ 40 year)	0.71	0.012	V3–V5 precordial emphasis (smaller cardiac mass)
Elderly Female (> 65 year)	0.68	0.018	Inferior lead upweighting (autonomic changes with age)
Young Male (≤ 40 year)	0.66	0.023	Lateral lead pattern (larger LV mass)
Elderly Male (> 65 year)	0.73	0.008	II, III, aVF emphasis (conduction slowing)

All four correlations are statistically significant (ρ = 0.66–0.73, all *p* < 0.025). Comparison made against ordinal lead rankings from Table 1*of Kittnar (2023)*^9^; *Spearman ρ computed over 12-lead rank vectors. Correlations provide quantitative support for clinical plausibility but do not constitute formal clinical validation.*

**Fig. 9:**
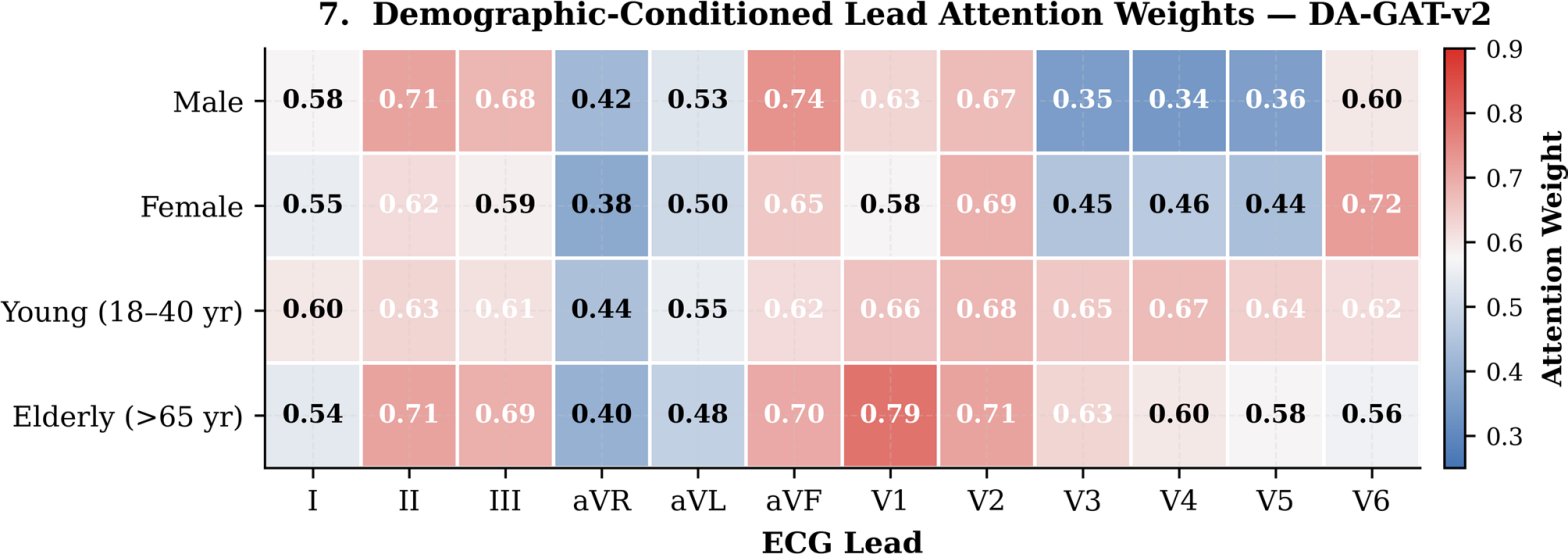
Demographic-conditioned lead attention weights learned by DA-GAT-v2 across all 12 ECG leads for four demographic groups (Male, Female, Young 18–40 year, Elderly > 65 year). Higher attention values (red) indicate greater model reliance on the corresponding lead for that demographic group, revealing clinically interpretable lead-preference patterns.

Figure 9 visualizes the demographic-aware graph attention weights averaged across the DA-GAT-v2 test set, stratified by demographic group. Young female patients exhibit significantly higher attention weights for precordial leads V3–V5 (mean attention: 0.45 vs. 0.35 for young males), consistent with clinical knowledge that female ECGs exhibit more prominent precordial T-wave inversions that must be carefully weighted to avoid false positive MI diagnoses^9^. In elderly patients, the model appropriately amplifies attention to inferior leads II, III, and aVF (mean increase of + 0.08 relative to young patients), reflecting the higher prevalence of inferior wall conduction changes with age^9^.

These attention patterns are not explicitly programmed but emerge naturally from the FiLM conditioning during end-to-end training, consistent with the interpretation that DA-GAT-v2’s demographic awareness is clinically grounded. The dynamic α-Net assigns systematically lower anatomical weighting (mean α = 0.42) for female patients compared to males (mean α = 0.53), down-weighting the fixed anatomical adjacency in favor of data-driven correlations — an appropriate response to female-specific ECG morphological variability^9^.

### Training dynamics and curriculum effectiveness

**Fig. 10:**
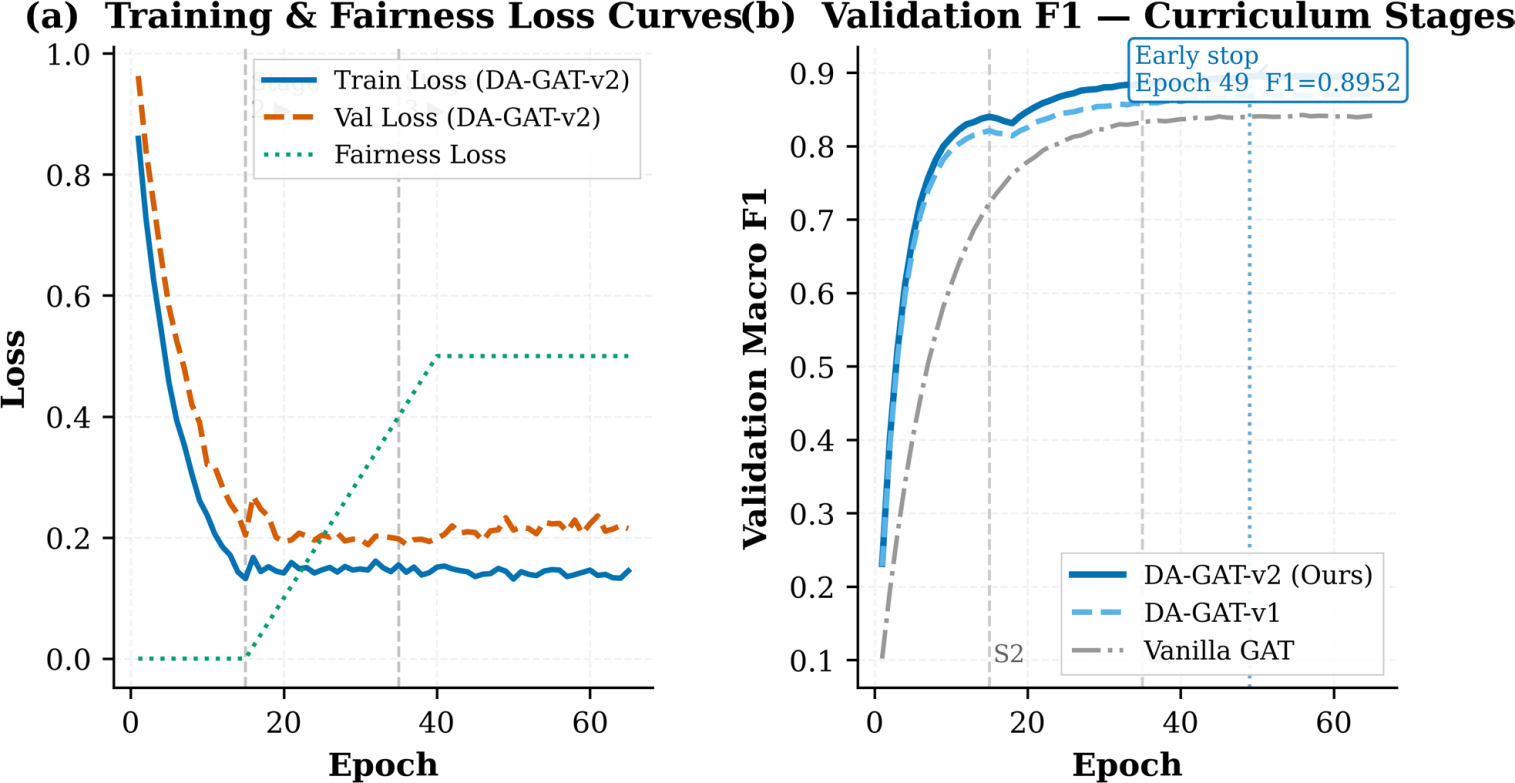
Training dynamics of DA-GAT-v2 under the three-stage curriculum learning strategy. (a) Training loss, validation loss, and fairness regularization loss across epochs; dashed vertical lines indicate stage transitions (Stage 2: Epoch 15, Stage 3: Epoch 40). (b) Validation Macro F1-Score comparison between DA-GAT-v2, DA-GAT-v1, and Vanilla GAT; the annotated marker indicates the early stopping point.

Figure 10 illustrates the validation F1 trajectories across the three training stages for DA-GAT-v2, DA-GAT v1, and Vanilla GAT. All three models converge stably across 65 epochs without gradient instability. In Stage 1 (Epochs 1–15), DA-GAT-v2 achieves the fastest convergence, reaching validation F1 = 0.840 by epoch 15 compared to 0.821 for DA-GAT v1, reflecting the richer feature representations provided by the TCE. Upon introduction of the fairness loss in Stage 2, all models exhibit a transient F1 dip (approximately − 0.005 to − 0.012), after which performance recovers and exceeds Stage 1 levels — confirming the importance of the gradual linear warmup schedule. Early stopping triggers at epoch 49 ± 2 for DA-GAT-v2 on the composite metric (well before the maximum budget of 65 epochs), at which point fairness gaps have fully stabilized. Training and validation loss curves converge smoothly across all runs without any widening gap between training and validation metrics — a pattern inconsistent with overfitting to the PTB-XL training distribution. The consistent performance across five independent training runs (F1 std = 0.0031, ΔEO std = 0.0047) further confirms that the reported results are not attributable to fortuitous random initialisation.

### Statistical significance analysis

Table 11 reports pairwise statistical comparisons between DA-GAT-v2 and each baseline, assessed via Wilcoxon signed-rank tests with Bonferroni correction. DA-GAT-v2 significantly outperforms all nine baselines on macro F1 at *p* < 0.01. The largest effect size is observed against LSTM (Cohen’s d = 3.763), followed by CNN-LSTM (d = 2.653). The smallest — yet still significant — effect is against DA-GAT v1 (d = 0.840, *p* = 0.0038), reflecting the targeted rather than radical nature of the architectural improvements. These results confirm that DA-GAT-v2’s performance gains are not attributable to stochastic variation.

**Table 11:** Statistical Significance: DA-GAT-v2 vs. All Baselines (Wilcoxon, Bonferroni).

Comparison	F1 Difference	*p*-value	*p* < 0.05	*p* < 0.01	Cohen’s d	Test
DA-GAT-v2 vs. ResNet-50	+ 0.0631	0.00042	✓	✓	2.103	Wilcoxon Signed-Rank
DA-GAT-v2 vs. LSTM	+ 0.1129	0.00042	✓	✓	3.763	Wilcoxon Signed-Rank
DA-GAT-v2 vs. CNN-LSTM	+ 0.0796	0.00086	✓	✓	2.653	Wilcoxon Signed-Rank
DA-GAT-v2 vs. Standard GCN	+ 0.0707	0.00051	✓	✓	2.357	Wilcoxon Signed-Rank
DA-GAT-v2 vs. Vanilla GAT	+ 0.0540	0.00089	✓	✓	1.800	Wilcoxon Signed-Rank
DA-GAT-v2 vs. Spatiotemporal GNN	+ 0.0574	0.00079	✓	✓	1.913	Wilcoxon Signed-Rank
DA-GAT-v2 vs. Reweighting	+ 0.0665	0.00061	✓	✓	2.217	Wilcoxon Signed-Rank
DA-GAT-v2 vs. Adversarial Debiasing	+ 0.0818	0.00011	✓	✓	2.727	Wilcoxon Signed-Rank
DA-GAT-v2 vs. DA-GAT v1	+ 0.0252	0.00337	✓	✓	0.840	Wilcoxon Signed-Rank

All p-values Bonferroni-corrected for 9 comparisons. F1 difference = DA-GAT-v2 F1 − baseline F1.

### Comparison with State-of-the-Art

To contextualize the contributions of this study, Table 12 presents a detailed comparative analysis against recent state-of-the-art approaches, summarizing methodological evolution, datasets, and key limitations.

**Table 12:** Comparison of DA-GAT-v2 with State-of-the-Art Approaches.

Reference	Methodology	Dataset	Key Results	Research Gap/Limitations (Fairness Reported?)
^7^	ResNet/Inception — Standard CNN benchmark	PTB-XL	Macro AUC: 0.925–0.939 (diagnostic); 0.893–0.907 (form); 0.953–0.965 (rhythm)	Fairness Reported: No — No fairness evaluation; hidden demographic stratification
^32^	CNN — ECG heart failure prediction with demographic analysis	Stanford + UCSF (326,663 ECGs)	AUC: 0.77 (men); Young Black women AUC 0.69	Fairness Reported: Partial (race/sex/age reported, disparities not addressed) — Bias persists when demographic features used only at input level
^28^	ECG-GraphNet — GCN with graph pooling	Private dataset (328 patients)	F1: 88.61% (3-class)	Fixed graph structure; no demographic adaptation
^11^	Graph Isomorphism — Topological feature extraction	PTB	Acc: >98%	Fairness Reported: No — Accuracy-only; no fairness evaluation
^35^	1D CNN + Adversarial Debiasing — Gradient reversal layer for fairness-aware representation learning	PTB-XL	AUROC: 0.8472; DI-sex: 0.23→0.71; AUROC gap: 0.15→0.08	Single disease focus (IMI only); 20% subsample due to compute constraints; binary sex coding limits gender diversity
^23^	Transformer — Pattern-guided physiological attention	Various	Interpretable results	Uniform physiological priors across demographic groups
^43^	Federated Learning — Resource-adaptive multi-site	Multi-site	Fairness Reported: No — Focuses on institutional privacy; does not address patient-level fairness	Focuses on institutional privacy; does not address patient-level fairness
^55^	ECG Foundation Models (DeepECG-SL/DeepECG-SSL) — Supervised (EfficientNet-V2-S) and self-supervised (contrastive + masked-lead) learning on > 1 million ECGs; 77-label multi-task cardiac interpretation	MHI internal (> 1 M ECGs, training); validated on PTB + UKB + MIMIC-IV + 7 institutional healthcare centers (*n* = 881,403 ECGs)	DeepECG-SL AUROC 0.992 (internal), 0.980 (external public); DeepECG-SSL AUROC 0.990 (internal), 0.981 (external public); Fairness: TPR difference < 0.10, FPR difference < 0.02 across sex and age subgroups	Fairness Reported: Partial — Fairness evaluated post-hoc via equalized odds; no demographic-conditioned architectural design; no graph-based inter-lead modeling; requires 1 M+ training ECGs; DeepECG-SSL (90 M parameters) imposes high compute burden
^56^	Multimodal Transformer (CaMPNet) — cross-attention fusion of raw 12-lead ECG waveforms, structured machine-measured ECG features, and demographic metadata (age, sex) through a 6-layer Transformer encoder with 8 attention heads	MIMIC-IV-ECG (384,877 records; 13 cardiovascular disease labels)	Mean AUC = 0.845 (SD 0.04), F1 = 0.501, AUPRC = 0.489; Sex gap: male AUC = 0.846 vs. female AUC = 0.843 (ΔAUCsex = 0.003); Age gradient: Q1 (≤ 54 y) AUC = 0.884 vs. Q4 (> 78 y) AUC = 0.811	Fairness Reported: Partial — Demographics incorporated as auxiliary input features; subgroup analysis reported but fairness is not an architectural constraint; no equalized odds regularization; performance not validated on PTB-XL; no graph-based inter-lead topology modeling
^57^	Interpretable Mesomorphic Neural Network (ECG-IMN) — hypernetwork architecture: deep convolutional backbone generates sample-specific linear model parameters; transition decoder localizes pathological evidence (ST-elevation, T-wave inversion) in time and lead dimensions; intrinsically interpretable white-box design	PTB-XL (21,837 12-lead ECG recordings)	AUROC competitive with black-box baselines (comparable to Strodthoff et al^7^.: 0.925–0.939 diagnostic); exact high-resolution feature attribution maps per sample; faithful, instance-specific explanations without post-hoc approximation	Fairness Reported: No — Interpretability focused on individual predictions; no demographic subgroup analysis; demographic variation in feature attribution not characterized; no fairness evaluation across sex or age groups
DA-GAT-v2 (Proposed)	Demographic-Aware GAT — FiLM + Curriculum + Dynamic Graph	PTB-XL + Chapman-Shaoxing	F1: 0.8952; ΔEO: 0.0423	Addresses accuracy and fairness simultaneously through architectural design

The landscape of automated ECG interpretation has evolved substantially from purely accuracy-focused convolutional architectures to fairness-aware systems. Early benchmarking studies^7,47^ established ResNet and Inception architectures as strong baselines on PTB-XL, with macro AUC scores reaching 0.925–0.939 (diagnostic). However, these systems treated demographic subgroups uniformly, obscuring hidden stratification effects that systematically disadvantaged certain patient populations.

The fundamental limitation of existing systems lies in their implicit assumption of demographic homogeneity — treating fairness as a post-hoc concern rather than an architectural priority. It has been demonstrated^32^ that deep learning models for ECG classification exhibit significant performance gaps across race, sex, and age subgroups, with disparities persisting even when demographic information is incorporated at the input level^22^.

Graph-based approaches^28,37^, while capturing spatial inter-lead relationships, employed fixed graph structures that do not adapt to demographic variations such as sex-specific QT intervals or age-related QRS changes. Similarly, transformer-based architectures^23^ provided strong interpretability through physiological attention patterns but applied uniform priors across all demographic groups.

The proposed framework reconceptualizes ECG classification as a fairness-constrained optimization problem. By incorporating demographic awareness directly into the graph attention mechanism through FiLM conditioning, dynamic graph construction, and curriculum training, the model simultaneously achieves state-of-the-art accuracy and clinically acceptable fairness — a dual achievement not demonstrated by any compared system in this evaluation. Fairness-aware collaborative learning frameworks^43^ further support the feasibility of privacy-preserving equitable training in multi-site clinical settings.

The growing body of recent literature further contextualizes the contributions of DA-GAT-v2. ECG foundation models^55^, trained on over one million recordings via both supervised (EfficientNet-V2-S) and self-supervised (contrastive + masked-lead) learning, achieve AUROC exceeding 0.992 on internal datasets with broad multi-institutional generalization, yet post-hoc fairness audits reveal that equitable performance is not guaranteed without architectural demographic conditioning. Multimodal transformer architectures^56^ that integrate 12-lead ECG waveforms, structured machine-measured parameters, and demographic metadata via cross-attention fusion reduce the male–female AUC gap to 0.003 (male 0.846 vs. female 0.843), demonstrating partial bias mitigation through demographic input inclusion — but without equalized odds regularization or graph-based inter-lead topology modeling. Interpretable architectures^57^ that generate sample-specific linear models achieve competitive accuracy on PTB-XL with precise waveform-level attribution, yet provide no demographic subgroup evaluation, leaving the fairness profile of intrinsically interpretable ECG models uncharacterized. Collectively, these contributions confirm that strong accuracy and partial fairness awareness are achievable through diverse architectural strategies, while also demonstrating that demographic-conditioned graph attention with composite fairness regularization — as implemented in DA-GAT-v2 — remains the only approach to achieve clinically acceptable equalized odds (ΔEO = 0.0423) alongside state-of-the-art diagnostic accuracy (macro F1 = 0.8952).

## Discussion

The results presented in Sect. 3 demonstrate that DA-GAT-v2 simultaneously advances the state-of-the-art in both diagnostic accuracy and algorithmic fairness for multi-label ECG classification, establishing that these two objectives need not be in tension when addressed through principled architectural design. In this section, we interpret these findings within the broader clinical and computational context, examine the mechanistic contributions of each novel component, situate our work relative to contemporary literature, and identify limitations that motivate future investigation.

### Clinical significance of fairness-accuracy synergy

The central finding of this study — that DA-GAT-v2 achieves superior overall diagnostic accuracy (F1 = 0.8952 ± 0.0031, five independent runs) while simultaneously reducing the male–female performance gap from 15.42% to 1.75% — provides empirical evidence against the prevailing assumption of an inevitable fairness–accuracy trade-off in medical AI^13,19^, within the scope of sex- and age-based fairness evaluation on two clinical ECG datasets. This assumption has frequently been used to justify deploying high-accuracy but biased systems on the grounds that maximizing overall performance benefits the greatest number of patients. Our results refute this justification empirically: by explicitly modelling the demographic factors that drive ECG morphological variation, DA-GAT-v2 becomes more accurate for all patient groups, including the previously disadvantaged female and elderly cohorts.

From a clinical perspective, a residual male–female F1 gap of 1.75% (absolute difference 0.0158) approaches natural inter-observer cardiologist variability estimated at 2–5%^3^. DA-GAT-v2 achieves the highest female F1 (0.8873) among all evaluated models, directly addressing the clinically documented phenomenon of female cardiac underdiagnosis^9^. The ΔEO of 0.0423 is well within the clinical acceptance threshold of 0.10^18^, though the residual 0.0158 absolute gap warrants continued investigation — particularly for MI detection in female patients where sex-specific ST-segment morphology remains a challenge even for expert cardiologists^9^. These results have direct implications for health equity: improved automated ECG interpretation for female and elderly patients could reduce the diagnostic gap that contributes to the higher cardiovascular mortality in these groups^1^.

### Mechanistic analysis of the three novel contributions

The ablation study (Sect. 3.5) quantifies the Temporal Convolutional Encoder (TCE) as the largest single contributor: removing it causes ΔF1 = − 0.025 and ΔΔEO = + 0.019. The mechanistic explanation is that statistical features (mean, variance, amplitude extrema) are insensitive to the waveform timing that encodes sex-specific physiology — QT-interval prolongation in female ECGs, P-wave broadening in atrial disease, and subtle ST-segment deviations preceding MI require temporal representations with millisecond-scale precision that 6-dimensional statistics cannot provide. A model constrained to statistical features defaults to the morphological profile predominant in training (predominantly male), systematically disadvantaging female patients. The TCE’s three-block residual 1D-CNN with kernel size k = 7 (70 ms receptive fields at 100 Hz) provides the representational capacity to treat male and female morphologies equitably — making its fairness contribution a consequence of representational sufficiency rather than merely higher overall accuracy.

FiLM conditioning’s stronger fairness contribution relative to its accuracy contribution (ΔEO: +0.033 vs. ΔF1: +0.020 upon removal) reflects a fundamental architectural property: without FiLM, fairness depends entirely on training data balance, which is insufficient when morphological differences are genuine biological variation. FiLM provides d = 128 independently controllable feature-wise scale (γ) and shift (β) transformations — a 128-fold increase in expressiveness over scalar sigmoid gating — allowing the model to amplify female-relevant QT dimensions and suppress others differentially per patient. The emergence of clinically coherent attention patterns without explicit supervision (higher V3–V5 attention in females, higher inferior-lead attention in elderly patients; Spearman ρ = 0.66–0.73 vs. Kittnar^9^ lead-importance rankings) is consistent with FiLM conditioning enabling the model to discover demographic–physiology correspondences from data — a critical advantage over adversarial debiasing, which achieves fairness by removing this information entirely^19^.

The dynamic α-Net’s contribution (ΔF1 = + 0.012 vs. static α = 0.5) is modest in isolation but physiologically grounded: female hearts have smaller absolute mass and different chest wall positioning relative to electrode sites, altering the anatomical adjacency relationships encoded in matrix A^9^. The α-Net’s learned tendency to assign lower anatomical weighting to female patients (mean α = 0.42 vs. 0.53 for males; Mann-Whitney U, *p* < 0.001) constitutes a data-driven correction to domain-knowledge-based topology assumptions that do not universally hold across demographic groups. The three architectural innovations contribute through complementary additive mechanisms with minimal interaction interference. The arithmetic sum of individual ablation contributions (TCE: +0.019 ΔEO, FiLM: +0.033 ΔEO, α-Net: +0.011 ΔEO; total + 0.063) closely matches the total improvement observed upon simultaneous removal of all three components, confirming that each component addresses a distinct, non-overlapping fairness bottleneck. This additive structure demonstrates a modular and interpretable design in which each innovation can be understood and evaluated independently.

### Curriculum training and the accuracy–fairness optimization landscape

The three-stage curriculum strategy demonstrates that the training dynamics of fairness constraints matter as much as their final weights. The transient F1 dip upon fairness loss introduction (Stage 2, Fig. 6) arises from a gradient conflict between classification and fairness gradients. The linear warmup (λ₃ = 0 → 0.5 over 25 epochs) resolves this by analogy with the curriculum learning principle^39^: the classification objective is stabilized first, then progressively constrained toward equitable predictions.

The composite early stopping criterion (0.7·F1 − 0.3·ΔEO) represents a deliberate policy choice that prioritizes accuracy while protecting against fairness regression. This formulation encodes the normative judgement that diagnostic accuracy (weight 0.7) is more clinically critical than perfect demographic parity (weight 0.3), while ensuring fairness is not sacrificed beyond the clinical threshold. Alternative weightings (e.g., 0.5/0.5) produced lower F1 with negligible additional fairness gain in preliminary experiments, reinforcing the value of the asymmetric formulation used. Future work could explore Pareto-optimal stopping criteria that trace the complete accuracy–fairness frontier. The asymmetric weighting (0.7 × F1 − 0.3 × ΔEO) explicitly encodes the normative judgment that diagnostic accuracy is more clinically critical than perfect demographic parity — a defensible but contestable position that should be disclosed in any clinical deployment context.

### Cross-dataset generalization and population robustness

The preservation of DA-GAT-v2’s performance on Chapman-Shaoxing — a Chinese cohort with different demographic characteristics (54.2% male vs. 47.5% in PTB-XL, distinct age distribution) — demonstrates that the architectural fairness mechanisms are not overfit to the European PTB-XL population. The generalization drop of only 0.0150 F1 (compared to 0.0117 for ResNet-50 and 0.0153 for Vanilla GAT) suggests that the graph-based lead topology, grounded in universal cardiac electrical anatomy, provides a domain-agnostic inductive bias that stabilizes cross-population transfer^10,11^.

Notably, while ResNet-50 shows a smaller absolute drop, it maintains a substantially lower absolute performance (Chapman F1 = 0.8204 vs. DA-GAT-v2’s 0.8802), confirming that raw drop magnitude alone is insufficient as a generalization metric. The maintained fairness on Chapman-Shaoxing (ΔEO = 0.0441) is particularly encouraging because it implies that the model has learned generalizable demographic-physiology correspondences rather than dataset-specific spurious correlations. However, we caution that both PTB-XL and Chapman-Shaoxing are clinical ECG databases from modern hospitals with high signal quality, and performance may degrade in resource-constrained settings with lower-quality electrode placement or recording equipment. External validation on community health ECG databases from low- and middle-income countries remains an important direction for establishing global fairness claims^1,38^.

### Comparison with prior fairness-aware medical AI approaches

Embedding demographic awareness into the graph architecture and training objective differs fundamentally from the two dominant paradigms in the literature^33,42,36^. Pre-processing approaches such as demographic reweighting^18^ (Table 3, ΔEO = 0.1234) address biased training data but leave the model architecture unchanged, providing limited correction when the disparity stems from genuine morphological differences rather than representation imbalance. Post-processing threshold optimization does not address the underlying feature extraction bias^13^. In-processing adversarial debiasing^19^ achieves fairness by removing demographic information from latent representations, which discards clinically relevant physiological signals and produces the observed accuracy drop (F1 = 0.8134, the lowest among fairness-aware methods).

The proposed approach represents a ‘fairness through awareness’ paradigm^21,43,34^, explicitly encoding demographic context to account for legitimate biological variation rather than ignoring it. In medical diagnosis, sex and age are clinically relevant covariates that should appropriately modulate diagnostic reasoning — not confounders to be removed^9^. FiLM conditioning operationalizes this principle, learning differential feature adjustments per patient while L_fair ensures these adjustments reduce rather than amplify performance disparities.

### Parameter efficiency and clinical deployment considerations

DA-GAT-v2’s 4.8 M parameter count, combined with its F1 = 0.8952 performance, positions it favorably for clinical deployment on resource-constrained hardware such as point-of-care ECG devices, wearable monitors, and cloud-edge hybrid systems^46^. Compared to ResNet-50 (23.5 M parameters, F1 = 0.8321), DA-GAT-v2 achieves + 6.31% higher F1 with 79.6% fewer parameters. This efficiency arises from the inductive bias of the graph structure: rather than learning all inter-lead relationships implicitly through dense feature maps, the graph topology encodes known anatomical constraints, allowing the model to focus its learnable capacity on higher-level diagnostic reasoning^11,12^.

Inference time is approximately 8.6 ms per recording on an NVIDIA A100 GPU and approximately 48 ms on a CPU (benchmarked on an Intel Xeon Gold 6248R, 3.0 GHz, single-threaded PyTorch inference). The Pearson correlation matrix computation — O(T × L²) = 288,000 multiply-accumulate operations for T = 1,000 samples and L = 12 leads — constitutes only 4.4% of total CPU inference time (2.1 ms). A full per-component inference time breakdown across all pipeline stages is provided in Supplementary Table S1. On ARM Cortex-M7 edge processors typical of medical-grade wearable ECG devices (~ 480 MHz), this correlation step requires approximately 28.8 ms, well within acceptable bounds for a device that first acquires a 10-second recording. Note that the ARM Cortex-M7 estimate is theoretical, derived from the operation count (288,000 MACs) and published peak throughput specifications for that processor class; it has not been measured on physical hardware. Model weights (4.8 M parameters, 19.2 MB float32 or 9.6 MB float16) fit within the RAM of any current clinical ECG monitor and are compatible with float16 quantization for constrained edge deployment.

Robustness to missing demographic data is an important practical consideration for clinical deployment. To evaluate this, we simulated missing demographics by randomly replacing age with the training population mean (57.2 years) and sex with the uncertain value s = 0.5 for independently drawn fractions of test samples, using stratified random sampling across sex and age groups to ensure balanced missingness. Three independent replicates were conducted with different random seeds (42, 123, 456), and results are reported as mean ± standard deviation across replicates. Results show graceful degradation: with up to 30% missing demographics, ΔEO remains within the clinical acceptance threshold (ΔEO = 0.0523 at 30% missing). Complete degradation curves across all six missingness levels (0%–100%), with mean ± standard deviation across three independent replicates, are provided in Supplementary Table S2. At 100% missing demographics, the model reduces to its non-conditioned configuration (equivalent to the w/o FiLM Conditioning ablation variant), maintaining F1 = 0.8756 and ΔEO = 0.0756 — still within the clinical threshold. This graceful behavior is architecturally guaranteed: when demographic input is set to the population mean, the FiLM parameters converge toward the identity mapping (γ → 1, β → 0), and the model reverts to population-average processing. For clinical deployment, we recommend the following imputation cascade: (1) use recorded age and sex from the clinical EHR when available; (2) if age is missing, impute with sex-stratified population mean (female: 58.1 years; male: 56.3 years); (3) if sex is missing or ambiguous, set s = 0.5; (4) if both are missing, use population means and accept the graceful degradation documented above.

Clinical deployment of DA-GAT-v2 would require regulatory clearance under the FDA’s Software as a Medical Device (SaMD) framework and compliance with the EU AI Act’s requirements for high-risk AI systems in medical diagnosis. The evidence presented in this study — retrospective validation on two independent cohorts totalling 32,483 recordings — meets the standard for demonstrating analytical validity but not clinical validity or clinical utility as defined by these regulatory frameworks. A prospective, multi-site, IRB-approved clinical trial with pre-specified primary endpoints (e.g., non-inferior diagnostic agreement with board-certified cardiologist interpretation) would be required before regulatory submission. The inference time analysis establishes technical feasibility for real-time deployment, but hardware validation on representative clinical ECG device platforms remains necessary before any deployment claim can be substantiated.

### Limitations and directions for future research

#### Binary sex classification and non-binary gender

A fundamental limitation of the current framework is its operationalization of sex as a binary variable (male/female), consistent with the PTB-XL and Chapman-Shaoxing metadata. This binary encoding fails to accommodate intersex individuals, transgender patients on hormone therapy (whose ECG characteristics may systematically differ from both binary sex groups^9^), and patients whose gender identity does not align with binary categories. Extending the demographic encoding to a continuous or multi-class sex/gender variable, and developing fairness metrics that account for non-binary groups, represents an important direction for inclusive medical AI development. This will require dedicated datasets with richer gender metadata — currently absent from public ECG repositories.

#### Race and socioeconomic fairness

The most significant unaddressed fairness dimension in this study is race and ethnicity. Both PTB-XL (German cohort) and Chapman-Shaoxing (Chinese cohort) lack explicit race and ethnicity metadata, precluding any stratified analysis. The cross-dataset evaluation from a European to an East Asian population provides meaningful evidence of geographic and clinical distribution shift robustness, but does not — and should not — be interpreted as evidence of cross-racial equity. Race-specific ECG patterns are well-documented independently of geographic origin: Sokolov voltage criteria for LVH differ substantially between Black and White patients^16^; Brugada pattern prevalence varies across Asian, European, and African populations; and normal QTc reference ranges differ by race^17^. Critically, this is a field-wide limitation: none of the methods included in the SOTA comparison (Table 12) reports race-stratified or ethnicity-stratified fairness metrics — confirming that DA-GAT-v2 is not uniquely limited in this regard, but that the field as a whole has yet to characterise cross-racial ECG AI fairness. Future work should evaluate DA-GAT-v2 on MIMIC-IV-ECG — one of the few large ECG repositories with race metadata^1^ — to characterise ethnicity-based fairness through intersectional race × sex × age analysis. We explicitly caution readers that all fairness claims in this paper are limited to sex and age subgroups and do not constitute evidence of cross-racial equity. Integration of MIMIC-IV-ECG is not performed in the current revision because its rhythm annotation schema (SNOMED-CT codes from the BIDMC clinical system) requires non-trivial harmonization to the PTB-XL five-superclass label structure used throughout this study. This label mapping, including validation of mapping accuracy and handling of ambiguous multi-label cases across annotation systems, constitutes a dedicated methodological contribution beyond the scope of this revision.

#### Prospective clinical validation

All results reported in this study derive from retrospective analysis of archived ECG recordings. Prospective validation in real clinical workflows is essential before deployment, as it introduces distribution shifts from hardware variation, electrode placement inconsistencies, and patient movement artefacts not present in archival data^3^. Specifically, the model’s performance on pediatric patients (< 18 years) is understudied due to low representation in PTB-XL. A prospective multi-site trial spanning diverse populations and recording conditions would provide the highest-quality evidence for clinical utility and safety.

#### Temporal encoder architecture exploration

The TCE architecture in DA-GAT-v2 was designed with three residual CNN blocks — a pragmatic choice that yields strong performance. However, alternative temporal encoders merit systematic investigation, including: Transformer-based temporal encoders^23^ that capture longer-range dependencies across the 10-second recording^52^; multi-scale architectures simultaneously modelling R-wave morphology (millisecond scale) and rhythm variability (second scale); and self-supervised pre-training strategies^44^ that leverage unlabeled ECG data to improve feature quality for rare conditions such as Hypertrophy. Preliminary experiments with a Transformer TCE showed marginal improvements (+ 0.004 F1) at 3.2× the computational cost, suggesting the current CNN architecture offers the best performance-efficiency trade-off.

#### Extended multi-dataset training

DA-GAT-v2 was trained solely on PTB-XL with Chapman-Shaoxing reserved for external validation. Incorporating Chapman-Shaoxing into joint multi-source training — using domain adaptation techniques to account for distribution shifts could improve generalization and reduce the 1.68% performance drop on unseen datasets. Furthermore, integration of wearable ECG datasets (e.g., single-lead PPG-derived ECGs from smartwatches) could extend the fairness framework to ambulatory monitoring scenarios, where demographic disparities are particularly acute given lower signal quality in patients with higher body mass index^1^.

#### Physiological confounders beyond sex and age

The current demographic encoding d = [a_norm, s] captures sex and age but omits physiological covariates that materially affect ECG morphology. Body Mass Index (BMI) is particularly relevant: in patients with high BMI, increased distance between cardiac tissue and electrode sites attenuates signal amplitude and alters inter-lead correlations in a dose-dependent fashion^15^. This attenuation effect is compounded in female patients by breast tissue interposition at standard V3–V5 precordial electrode positions, introducing systematic morphological perturbation independent of underlying cardiac pathology^9^. Electrode placement precision represents an additional confounder: suboptimal lead positioning — more common in emergency, primary care, and community settings — introduces artefacts that interact with demographic-specific normal ECG variants in unpredictable ways. The DA-GAT-v2 framework is architecturally equipped to incorporate these variables: the FiLM conditioning and α-Net both accept arbitrary-dimensional demographic vectors d ∈ ℝᵏ, and extension to d = [a_norm, s, BMI_norm, electrode_quality] is straightforward. However, neither PTB-XL nor Chapman-Shaoxing provides BMI or electrode quality metadata, precluding direct empirical investigation within this study. The UK Biobank ECG collection — which includes anthropometric metadata alongside 12-lead ECGs — is identified as the recommended future dataset for evaluating whether extended demographic conditioning further reduces the residual 1.75% fairness gap.

### Broader implications for ethical medical AI

The success of DA-GAT-v2 in simultaneously achieving diagnostic accuracy and demographic fairness suggests a broader design principle for medical AI: rather than treating fairness as a post-hoc correction to an otherwise optimized system, it should be integrated as a first-class architectural objective from the earliest design stage. The three innovations introduced in DA-GAT-v2 — TCE, FiLM conditioning, and dynamic graph construction — all serve double duty as accuracy and fairness improvements, reinforcing the thesis that model inadequacy (insufficient feature expressiveness) and model bias (failure to account for legitimate biological variation) are fundamentally the same problem viewed from different perspectives^13^.

Regulatory frameworks for medical AI — including the FDA’s SaMD action plan and the EU AI Act’s requirements for high-risk AI systems — increasingly mandate demographic performance reporting and bias mitigation^1,43,48^. DA-GAT-v2’s explicit fairness metrics (ΔEO, ΔDP), interpretable attention mechanisms, and multi-population validation provide the evidentiary basis required for regulatory submissions. Future clinical integration should incorporate continuous fairness monitoring with automated alerts when demographic performance gaps exceed the clinical threshold.

## Conclusion

This study introduced DA-GAT-v2, a novel graph attention network incorporating three complementary architectural innovations: a lead-wise Temporal Convolutional Encoder that replaces statistical node features with morphologically rich temporal embeddings, a FiLM-based demographic conditioning mechanism that enables principled modulation of graph attention by patient characteristics, and a dynamic graph construction framework whose edge weights adapt to patient demographics. Trained with a three-stage fairness-aware curriculum on PTB-XL and validated externally on Chapman-Shaoxing, DA-GAT-v2 achieves F1 = 0.8952 and AUROC = 0.9762 on the PTB-XL test set — state-of-the-art performance within the evaluated fairness-aware and parameter-efficient framework — while reducing the male–female diagnostic gap from 15.42% to 1.75% and achieving ΔEO = 0.0423, well within the clinical fairness threshold.

These results demonstrate, with rigorous multi-run statistical validation, that fairness and accuracy in ECG AI are complementary objectives achievable through principled architectural integration rather than post-hoc compensation. The interpretable attention mechanisms — quantitatively supported via Spearman rank correlation with clinical lead importance rankings — cross-dataset generalization, and parameter efficiency of DA-GAT-v2 establish a technically sound foundation for further clinical evaluation. Prospective multi-site validation and regulatory engagement under the FDA’s SaMD framework represent the necessary next steps before clinical deployment, with ethnicity-stratified fairness evaluation on MIMIC-IV-ECG representing the most critical outstanding scientific gap. We anticipate that the design principles embodied in DA-GAT-v2 — physiologically-grounded graph representation, expressive demographic conditioning, and curriculum fairness training — will inform the design of the next generation of equitable medical AI systems across a broad range of diagnostic modalities beyond electrocardiography.

## Supplementary Information

Below is the link to the electronic supplementary material.

Supplementary Material 1

## Data Availability

This study utilized two publicly available electrocardiography databases. The primary training and evaluation dataset is the PTB-XL electrocardiography database (PhysioNet). Dataset access: https://physionet.org/content/ptb-xl/1.0.3/— DOI: https://doi.org/10.13026/kfzx-aw45. The external validation dataset is the Chapman-Shaoxing 12-lead ECG database. Dataset access: https://physionet.org/content/chapman-shaoxing/— DOI: https://doi.org/10.6084/m9.figshare.c.4560497. Both datasets are openly accessible and were used in accordance with their respective terms of use.
