# Supplementary material for: Demographic-aware temporal graph attention for fair and accurate cardiac abnormality detection in 12-lead ECG
Source: Sci Rep. 2026 Jun 4;16:17379. doi: 10.1038/s41598-026-54206-8 (PMC13237346; doi:10.1038/s41598-026-54206-8)
Supplement: Supplementary file 1 — Supplementary Material 1 [file 41598_2026_54206_MOESM1_ESM.docx]

**SUPPLEMENTARY MATERIAL**

Demographic-Aware Temporal Graph Attention for Fair and Accurate Cardiac Abnormality Detection in 12-Lead ECG

## Supplementary Table S1 — Per-Component Inference Time Breakdown

The table below decomposes the total per-recording inference time of DA-GAT-v2 into its six pipeline components, benchmarked on CPU (Intel Xeon Gold 6248R, 3.0 GHz, single-threaded) and GPU (NVIDIA A100). The Pearson correlation matrix — the component specifically queried by the reviewer — constitutes only 4.4% of total CPU inference time. The dominant computational cost is the Temporal Convolutional Encoder (59.4%), consistent with its role as a three-block residual 1D-CNN processing 12 leads in parallel.

| **Pipeline Component** | **CPU Time (ms)** | **GPU Time (ms)** | **% CPU Total** | **Notes** |
| --- | --- | --- | --- | --- |
| Signal preprocessing | 3.2 | 0.8 | 6.7% | Bandpass filtering + per-lead standardisation |
| Pearson correlation matrix | 2.1 | 0.3 | 4.4% | O(T×L²) = 288,000 MACs; T=1,000, L=12 |
| α-Net demographic weighting | 0.4 | 0.05 | 0.8% | 2-layer MLP, 2→16→1 architecture |
| TCE — 12 leads (parallel) | 28.5 | 5.2 | 59.4% | 3-block residual 1D-CNN; dominant compute step |
| GAT forward pass (2 layers) | 12.6 | 2.1 | 26.3% | Includes FiLM γ/β modulation per layer |
| Classification + aux heads | 1.2 | 0.1 | 2.5% | Sigmoid output + demographic regression head |
| **TOTAL per recording** | **48.0 ms** | **8.6 ms** | **100%** | **Single-threaded; Intel Xeon Gold 6248R (CPU) / NVIDIA A100 (GPU)** |
| Note. CPU benchmarks: Intel Xeon Gold 6248R, 3.0 GHz, single-threaded PyTorch inference. GPU benchmarks: NVIDIA A100. ARM Cortex-M7 (~480 MHz) theoretical estimate for the Pearson correlation step: ≈28.8 ms, derived from operation count (288,000 MACs) and published peak throughput specifications; not measured on physical hardware. Model weights: 4.8M parameters = 19.2 MB (float32) / 9.6 MB (float16). Compatible with float16 quantization for constrained edge deployment. | | | | |

## Supplementary Table S2 — Robustness to Missing Demographic Data

The table below reports DA-GAT-v2 performance across six levels of demographic data missingness (0% to 100%), evaluated on the PTB-XL test set (n = 3,226) using three independent random seeds. Missing demographics were simulated by replacing age with the training-set population mean and sex with s = 0.5. The model exhibits monotonic and graceful degradation: ΔEO remains within the clinical acceptance threshold (< 0.10) at all missingness levels including 100% (ECG-only fallback). The 100% missing row is architecturally equivalent to the 'w/o FiLM Conditioning' ablation (Table 8, main manuscript), confirming internal consistency.

| **Missing Fraction** | **F1 Macro (mean ± std)** | **ΔEO (mean ± std)** | **ΔF1 vs 0% missing** | **ΔΔEO vs 0% missing** | **Clinical Status** |
| --- | --- | --- | --- | --- | --- |
| **Missing %** | **3 seeds: 42, 123, 456** | **3 seeds: 42, 123, 456** | **Absolute change** | **Absolute change** | **Assessment** |
| **0% (complete — baseline)** | 0.8952 ± 0.0031 | 0.0423 ± 0.0047 | — | — | **Full performance — baseline** |
| 10% | 0.8931 ± 0.0033 | 0.0451 ± 0.0051 | −0.0021 | +0.0028 | Negligible degradation |
| 20% | 0.8907 ± 0.0035 | 0.0489 ± 0.0054 | −0.0045 | +0.0066 | Clinically acceptable |
| 30% | 0.8871 ± 0.0038 | 0.0523 ± 0.0059 | −0.0081 | +0.0100 | Within ΔEO < 0.10 threshold |
| 50% | 0.8812 ± 0.0044 | 0.0601 ± 0.0067 | −0.0140 | +0.0178 | Recommend imputation protocol |
| **100% (ECG-only fallback)** | 0.8756 ± 0.0050 | 0.0756 ± 0.0074 | −0.0196 | +0.0333 | **Graceful fallback — ΔEO < 0.10 ✓** |
| Note. Missing demographics simulated by replacing age with training-set population mean (57.2 years) and sex with s = 0.5 for independently drawn fractions of test samples, using stratified random sampling across sex and age groups to ensure balanced missingness. Three independent replicates conducted (seeds 42, 123, 456); results reported as mean ± std across replicates. Fallback value at 100% missing: d̄ = [0.572, 0.475] (min-max normalised age mean and proportion female from PTB-XL training partition). FiLM near-identity behaviour verified at d̄: mean \|γ − 1\| = 0.031, mean \|β\| = 0.028 across all 128 feature dimensions — confirming no subgroup bias is introduced. The 100% missing row corresponds exactly to the 'w/o FiLM Conditioning' ablation variant in Table 8 of the main manuscript (F1 = 0.8756, ΔEO = 0.0756). Clinical threshold: ΔEO < 0.10 [as defined in Section 2.9 of the main manuscript]. | | | | | |
